# Supplementary material for: Environmental and ecological controls of the spatial distribution of microbial populations in aggregates
Source: PLoS Comput Biol. 2022 Dec 19;18(12):e1010807. doi: 10.1371/journal.pcbi.1010807 (PMC9810174; doi:10.1371/journal.pcbi.1010807)
Supplement: S1 Appendix — Including the decision of the eco-interactions; explanation of discretization and methodology of diffusion-reaction equation; description of reaction term; details about shoving algorithm; and the algorithm of the integration process. (PDF) [file pcbi.1010807.s001.pdf]

Supporting Information

Environmental and ecological controls of the spatial distribution of microbial populations in aggregates

Eloi Martinez-Rabert[1]*, Chiel van Amstel[2], Cindy Smith[1], William T. Sloan[1], Rebeca Gonzalez-Cabaleiro[2]

[1] James Watt School of Engineering, Infrastructure and Environment Research Division, University of Glasgow, Advanced Research Centre, Glasgow, United Kingdom

[2] Department of Biotechnology, Delft University of Technology, Delft, Netherlands

*2424069M@student.gla.ac.uk

Table of contents

1. Decision of the selection of eco-interactions 2

2. Discretization of diffusion-reaction equation 3

1.1. Boundary conditions of simulation domain 4

1.2. Domain definition – diffusion and no-diffusion regions 6

2. Reaction term 9

2.1. Microbial activity 9

3. Shoving algorithm 10

3.1. Quadtree algorithm 11

4. Integration 15

5. References 17

1. Decision of the selection of eco-interactions

Considering *neutralism* [0,0], *competition* [–,–], *commensalism* [0,+], *mutualism* [+,+], *parasitism* [–,+] and *amensalism* [–,0] as the 6 main ecological interactions (Fig. 1), this study has focused on the three first cases (*neutralism*, *competition* and *commensalism*) and concurrence of *competition* and *commensalism*. Here, we stated the reasons of this decision:

- *Mutualism* [+,+]. Distinguishing cross-feeding (or *syntrophy*) and co-protection (or *symprostasy*) as two specific ecological interactions belonging to mutualism:
  - Cross-feeding (*substrate-related mutualism*). This ecological interaction has already been studied by Mitri, S. *et al.*, 2011 [1] and Momeni *et al*, 2013 [2], evaluating the influence of substrate(s) concentration and the presence of competition between microbial communities. Additionally, *mutualism* is one of the less frequent ecological interactions among culturable bacteria (only 5% of all assessed interactions among 20 soil bacteria across 40 carbon environments corresponded to a mutualistic interaction; study from Kehe *et al.*, 2021 [3]. As stated in Palmer and Foster, 2022 [4] – "*Negative interaction prevails, and cooperation, where two species both benefits, is typically rare*".
  - Co-protection (*inhibitor-related mutualism*). From an ecological perspective, the resource availability has a direct impact on interactions among species (e.g., a limited resource environment intensifies the competition for this resource). For this reason, the influence of substrate concentration has been evaluated in this study. Note that when inhibition of some community member is present in the system (either for presence of inhibitor in the environment, (*co)protection*; or produced by other community member, *amensalism*), the inhibitory influence is strengthened as higher is the concentration of inhibitor. Therefore, co-protection has an inverse substance-related trend to the selected ecological interactions (*neutralism, competition* and *commensalism*). For this reason, we decided to not include co-protection in this study.
- *Parasitism* [–,+]. One of the aims of this study is to study the combination of two ecological interactions in which one have a positive impact (as *commensalism*) and the other a negative impact (as *competition*). Due to *parasitism* has already a negative impact in one of the species, we decided to not include it in this study.

- *Amensalism* [–,0]. As (co)protection, *amensalism* has an inverse substance-related trend to the selected ecological interactions (*neutralism*, *competition* and *commensalism*). Moreover, *amensalism* has already a negative impact in one of the species. For these reasons, we decided to not include *amensalism* in this study.

2. Discretization of diffusion-reaction equation

To solve the diffusion-reaction equation (Eq 2; main manuscript), the implicit Crank-Nicolson method, which is unconditionally stable [5], is used to discretize in time the diffusion term. For the reaction term, an explicit forward Euler formula is used (Eq A1). This can be done, as the reaction process has a much slower time scale than the diffusion [6, 7].

$$\frac{\phi_{i,j}^{n+1} - \phi_{i,j}^{n}}{h_t} = \mathbb{D} \cdot \frac{1}{2}\left[\nabla^2 \phi_{i,j}^{n+1} + \nabla^2 \phi_{i,j}^{n}\right] + R(\phi_{i,j}^{n}) \quad n \in 1 ... N_t \tag{A1}$$

Where h_t refers to the time step, N_t to the total number of time steps and $\phi_{i,j}^{n}$ to substrate concentration in *node i,j* and time *n*. The Laplacian of Eq A1 (∇^2) is discretised in a two-dimensional space (x, y) using the central finite-difference method (Eq A2), where h is the grid size ($h = \Delta x = \Delta y$).

$$\nabla^2 \phi_{i,j}^{n} = \frac{\phi_{i-1,j}^{n} + \phi_{i+1,j}^{n} + \phi_{i,j-1}^{n} + \phi_{i,j+1}^{n} - 4\phi_{i,j}^{n}}{h^2} \tag{A2}$$

Eq A2 (also known as discrete Laplacian) is given as the following kernel (Eq A3), and Laplacian approximation is re-written in matrix from (Eq A4), where the convolution of $[L]$ and $\phi_{i,j}^{n}$ is denoted using the symbol *.

$$[L] = \begin{pmatrix} 0 & 1 & 0 \\ 1 & -4 & 1 \\ 0 & 1 & 0 \end{pmatrix} \tag{A3}$$

$$\nabla^2 \phi_{i,j}^{n} = \frac{1}{h^2}([L] * [\phi^n]) \tag{A4}$$

Where $[\phi^n]$ is the concentration of soluble compounds ($\phi_{i,j}^{n}$) defined in matrix form (Eq A5).

$$[\phi^n] = \begin{pmatrix} \phi_{1,1}^n & \phi_{1,2}^n & \cdots & \phi_{1,N_y}^n \\ \phi_{2,1}^n & \phi_{2,2}^n & & \vdots \\ \vdots & & \ddots & \vdots \\ \phi_{Nx,1}^n & \cdots & \cdots & \phi_{N_x,N_y}^n \end{pmatrix} \in M_{N_x \times N_y} \quad \text{(A5)}$$

For discrete and 2-dimensional variables (A and B), Eq A6 defines the convolution of A and B (i.e., A ∗ B). Convolution satisfies the distributive property, multiplicative identity, and associative property with scalar multiplication. These properties are essential to rearrange the diffusion-reaction equation properly.

$$A[x] * B[x] = \sum_{k=-\infty}^{+\infty} A[x] \cdot B[x-k] \quad \text{(A6)}$$

Then diffusion-reaction equation (Eq 2; main manuscript) can be re-written as a matrixes system (Eq A7), where ψ is a constant defined for each soluble component (Eq A8) and $[I_k]$ is the so-called *identity kernel* or *do-nothing convolution kernel* (Eq A9).

$$([I_k] - \psi \cdot [L]) * [\phi^{n+1}] = ([I_k] + \psi \cdot [L]) * [\phi^n] + R([\phi^n]) \cdot h_t \quad \text{(A7)}$$

$$\psi = \frac{\mathbb{D} \cdot h_t}{2 \cdot h^2} \quad \text{(A8)}$$

$$[I_k] = \begin{pmatrix} 0 & 0 & 0 \\ 0 & 1 & 0 \\ 0 & 0 & 0 \end{pmatrix} \quad \text{(A9)}$$

1.1. Boundary conditions of simulation domain

To solve Eq A7 in all simulation domain, it is necessary to define the boundary conditions of the problem. In this case, a single boundary condition type is defined – the concentrations at outside of the aggregate are defined by the conditions of the bulk liquid (Fig A1).

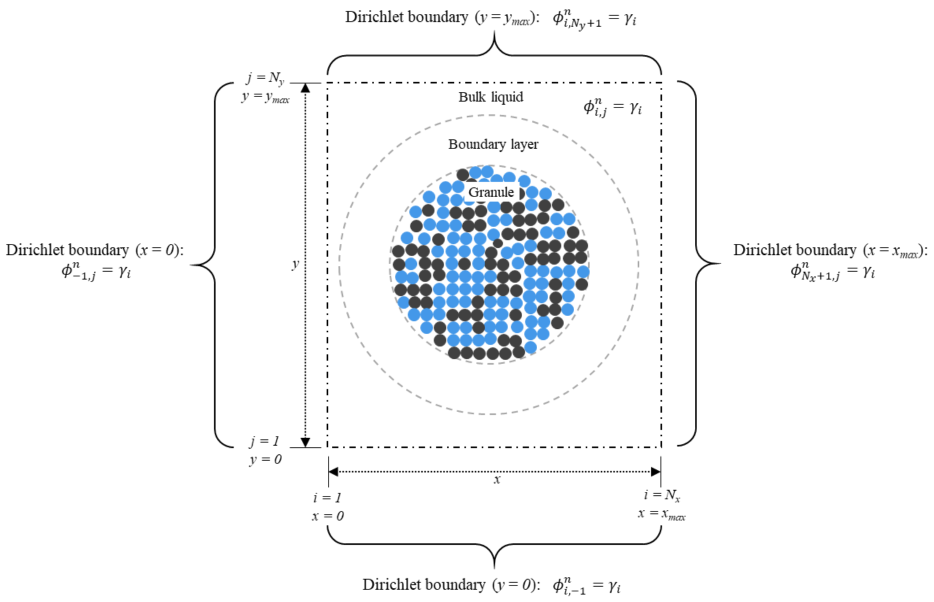

Fig A1. Considered boundary conditions

This is implemented by the Dirichlet (or first-type) boundary condition, in which is imposed the concentration of the bulk liquid (γ) at the extreme of the boundary layer (Eq A10) and bulk liquid.

$$\phi^n_{-1,j} = \phi^n_{N_x+1,j} = \phi^n_{i,-1} = \phi^n_{i,N_y+1} = \gamma \quad \text{(A10)}$$

To implement Dirichlet boundary condition in this system, first the region of the simulation domain is determined. Then, which nodes would belong to diffusion region (comprising boundary layer and granular region) are determined (see section 1.2). The concentration matrix $[\phi^n]$ is modified properly (Eq A11) – those nodes in which are considered diffusion region have the corresponding concentration value ($[\phi^n]$), and that ones in which are considered no-diffusion region have the boundary value (i.e., concentration of bulk liquid, γ).

$$[\phi^n]^\gamma = diffR \circ [\phi^n] + \neg diffR \cdot \gamma \quad \text{(A11)}$$

Where $diffR$ is a logical matrix with 1 (*true*) in those nodes of diffusion region, and 0 (false) in those nodes of no-diffusion region; $\neg diffR$ is the inverse of $diffR$. Then, the diffusion-reaction equation including the boundary conditions imposed over the simulation domain is written in Eq A12. For each soluble component and time iteration, Eq A12 is solved calculating ϕ^{n+1} using an efficient multigrid method (V-cycle) [8].

$$([I_k] - \psi \cdot [L]) * [\phi^{n+1}] = ([I_k] + \psi \cdot [L]) * [\phi^n]^\gamma + R([\phi^n]) \cdot h_t \quad \text{(A12)}$$

In this case, it is considered that the simulated aggregate grows in a continuous stirred tank reactor (CSTR), where substrate concentrations in the bulk liquid are changing due to the activity of microorganisms. The average microbial activity of the aggregate (as a representation of the activity of the whole reactor) is used to integrate the concentration of the soluble components in the bulk liquid of the reactor (S) through a mass balance (Eq A13).

$$\frac{dS}{dt} = \frac{1}{HRT} \cdot \left(S_{inf} - S\right) + R \qquad \text{(A13)}$$

Where *HRT* refers to the hydraulic time fixed in the reactor, $S_{inf,i}$ to the concentration in the influent and *R* to the reaction term considered in reactor. As mentioned, the reaction term (*R*) is calculated assuming the average of the reaction terms of all nodes (Eq A14).

$$R = \frac{\sum_{i=1,j=1}^{i=N_x,j=N_y} R_{i,j}{}^{n}}{N_x \cdot N_y} \qquad \text{(A14)}$$

The substrate concentrations in the influent are prefixed. For that, a dynamic HRT (Eq A15) is applied on the model to maintain the concentration of substrates constant.

$$HRT = \frac{(S_{inf} - S)}{R_{r,S}} \qquad \text{(A15)}$$

The new concentration *S* calculated with the integration of Eq A13 (using the build-in ode45 solver in MATLAB) updates the Dirichlet value (γ) included in Eq A11.

1.2. Domain definition – diffusion and no-diffusion regions

The diffusion region, where diffusion-reaction equation is solved (Eq A12), comprises the aggregate region and boundary layer. The aggregate region is basically the grid cells that hold bacteria, and boundary layer is the grid cells in the immediate vicinity of grid cells with bacteria. The thickness of boundary layer is selected by the user.

The distinction of the diffusion region starts with the detection of which nodes are potentially in this region, creating a preliminary diffusion region (Fig A2 a). For that, the minimum and maximum position of bacteria on aggregate in coordinate *x* and *y*, and boundary layer thickness are considered. The computational cost is reduced by using this preliminary diffusion region instead of the entire simulation domain.

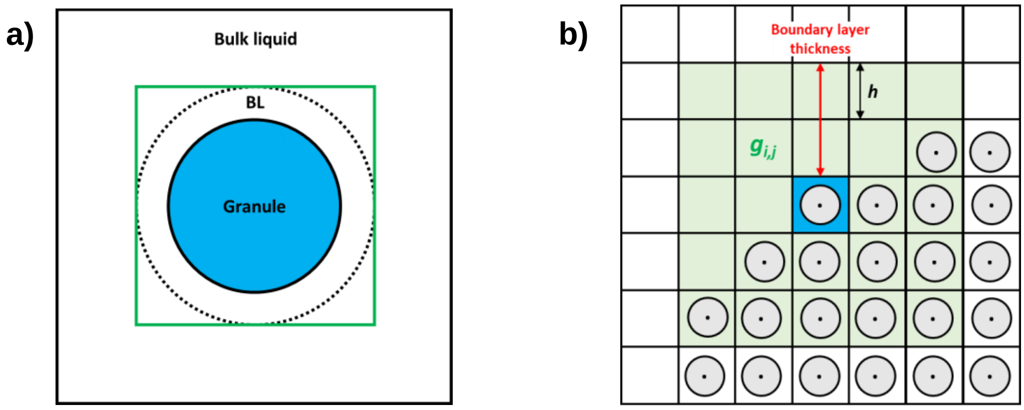

Fig A2. Detection of diffusion region. (A) Representation of preliminary diffusion region (green square). Legend: BL – boundary layer. (B) Neighbouring grid cells (green squares, $g_{i,j}$) of a specific boundary grid cell with bacteria (blue square) with potential to be included in diffusion region.

Then, grid cells that hold bacteria (at least one bacterium) are determined and included in diffusion region. Thus, it is defined the aggregate region. To recognise which grid cells are belonging to boundary layer region (and including them in diffusion region), the boundary grid cells with bacteria (i.e., the outermost grid cells of aggregate region) must be sought. An efficient way to find them is through the convolution of aggregate region matrix and *edge detection kernel* (Eq A16).

$$[K]_{ED} = \begin{pmatrix} -1/8 & -1/8 & -1/8 \\ -1/8 & 1 & -1/8 \\ -1/8 & -1/8 & -1/8 \end{pmatrix} \quad \text{(A16)}$$

Once found the boundary grid cells with bacteria, it is time to check whether the neighbouring grid cells ($g_{i,j}$) belong to boundary layer region and, thus, the diffusion region. By dividing the boundary layer thickness by grid size (h), the extent of the neighbouring grid cells with possibility to belong the boundary layer region is obtained (Fig A2 b).

For each boundary grid cell with bacteria, the neighbouring grid cells with potential are selected and evaluated whether they are actually in boundary layer region or not. If they are in it, they are included in diffusion region.

Finally, it is defined a focus region in which includes all diffusion nodes, has at least one bulk layer node at each side of diffusion nodes, and has an odd number of nodes in x and y coordinates (for efficient solution of diffusion-reaction equation [9]). The focus region establishes the region of the simulation domain where diffusion-reaction equation

is solved, and the pseudo-steady state is checked. The flowchart of diffusion region determination is presented in Fig A3.

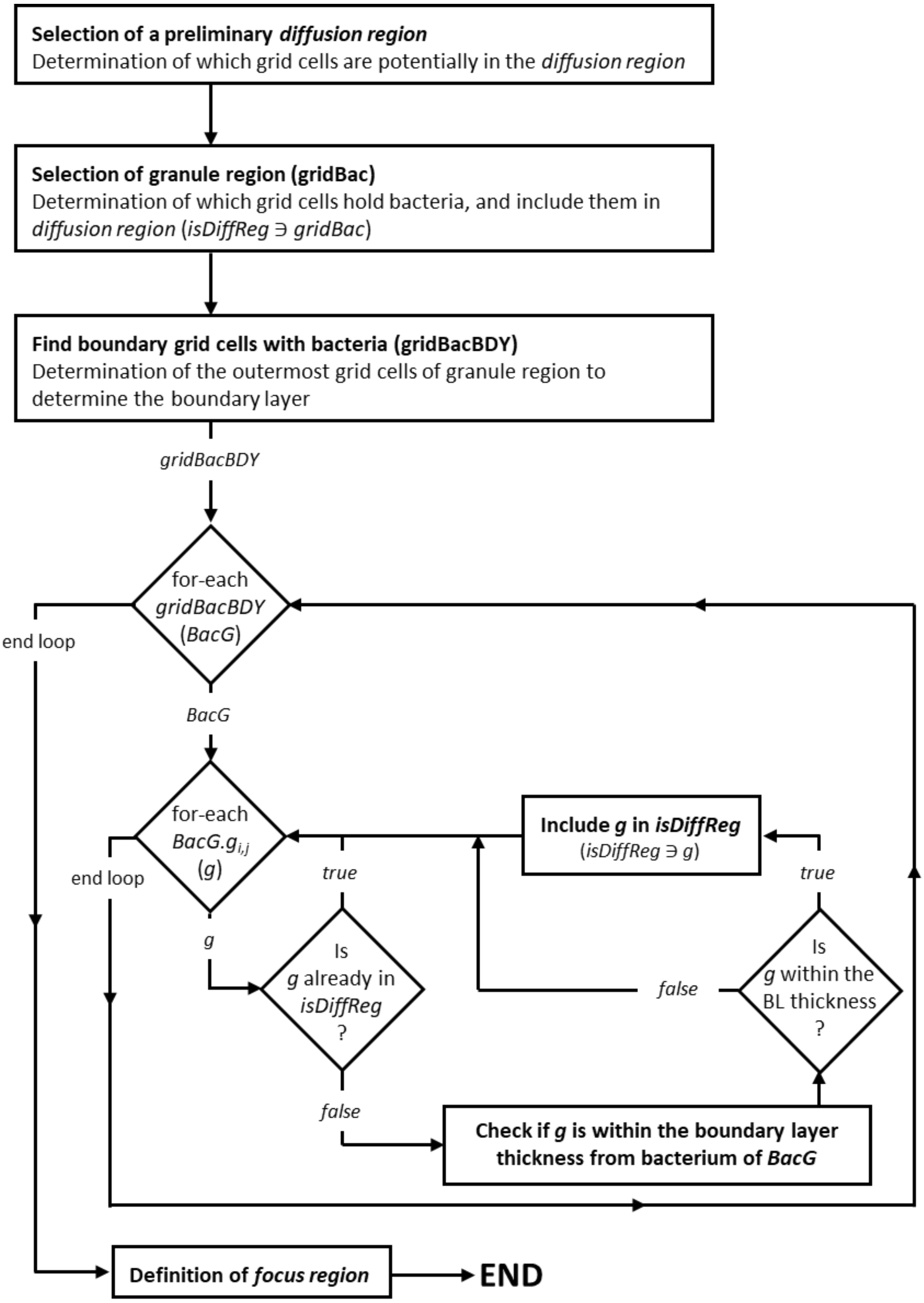

Fig A3. Algorithm of diffusion region determination

2. Reaction term

The matrix of reaction components $R([\phi^n])$ must be calculated and added to Eq A12. The reaction of the soluble components in this system is considered function of the microbial activity of the aggregate.

2.1. Microbial activity

The kinetics of microbes that grow in the aggregate are calculated function of the local conditions of the node where the cell is located inside the simulation domain. The kinetics parameters associated to each of the microbial species considered are included in S1 Table. But also, it is also needed to indicate the stoichiometry of the microbial species, in which will also define the corresponding eco-interaction (S2 Table). Eqs. A17 – A19 show examples of stoichiometries for anabolism (*Ana*), catabolism (*Cat*) and decay (*Dec*), where C_s and N_s refer to carbon and nitrogen sources, *eD* and *eA* to electron donor and acceptor and *X* to biomass considering an average formula of $C_1H_{1.8}O_{0.5}N_{0.2}$ [10, 11].

$$Ana \;=\; 1\cdot C_s + 0.2\cdot N_s \;+\cdots\rightarrow\cdots+\;1\cdot\;X \tag{A17}$$

$$Cat \;=\; \alpha\cdot\;eD\;+\;\beta\cdot\;eA\;\rightarrow\cdots \tag{A18}$$

$$Dec \;=\; 1\cdot\;X+\cdots\rightarrow\;1\cdot C_s + 0.2\cdot N_s \tag{A19}$$

In this case, it is assumed that:

(i) all bacteria have the same anabolic pathway;

(ii) carbon, nitrogen and other anabolic-related component are not limited, only *eD* and *eA* are limited.

The stoichiometry of the overall metabolism (*Met*) is calculated function of the catabolic and anabolic stoichiometries, and the growth yield (Y_{XS}, Eq A20). Moreover, it is assumed that all bacteria have the same value of Y_{XS} (see S2 Table).

$$Met \;=\; \frac{1}{Y_{XS}}\cdot Cat + Ana \tag{A20}$$

With the metabolic stoichiometry and the kinetic parameters (μ^{max}, K_S, K_I and b^{max}) for each of the microbial species present in the reactor, it is possible to calculate the growth rate (μ, Eq 3; main manuscript) of microbe. Then, through the stoichiometry and the mass of the microbe, the growth/decay of cell, substrate uptakes and product generations.

Eq A21 is the derivative of the mass of a microbe which is integrated in time using a forward Euler scheme; and Eq A22 computes the reaction term for a specific soluble component *s* due to the activity of the microbe *m*.

$$\frac{dX_m}{dt} = \mu_m^n \cdot X_m^n \quad \text{(A21)}$$

$$R_m^n = \frac{\mu_m^n \cdot \delta_{s,m}}{V_{xy}} \quad \text{(A22)}$$

Where X_m refers to the mass in moles of the cell *m*, $\delta_{s,m}$ to the stoichiometric coefficient of the substrate S for the cell *m* and V_{xy} to the volume of one node of the simulation domain.

The calculation of the reaction term for the soluble components is function of the position in the simulation domain (one reaction term per node for each soluble component). Therefore, Eq A23 is used to consider all microorganisms that are contributing to the reaction term of a specific soluble component in the *node i,j*.

$$R_{i,j}^n = \sum_{m=1}^{m=M_{i,j}} R_m^n \quad \text{(A23)}$$

Where $M_{i,j}$ refers to all microorganism that are in the *node i,j*. The reaction terms R_{ij}^n calculated by Eq A23 for all nodes of the simulation domain ($R([\phi^n])$) are included in the Eq A12 to calculate the concentration of each of the soluble components in all the simulation domain.

3. Shoving algorithm

To compute the shoving of the cells in the aggregate after cell growth and division, first the overlap between microorganisms is checked by Eq A25.

$$|\vec{v}| = \sqrt{(x_m - x_{m+1})^2 + (y_m - y_{m+1})^2} \quad \text{(A24)}$$

$$overlap = kDist \cdot (r_m + r_{m+1}) - |\vec{v}| \quad \text{(A25)}$$

Where $|\vec{v}|$ is the norm of the vector that links the centres of both cells *m* and *m+1* and $kDist$ is just a multiplier that allows adjustment of the minimal spacing between bacteria.

In this case, *quadtree algorithm* is applied to detect the *overlapping* between bacteria and, subsequently, the shoving of these is computed [12].

If the *overlap* value is bigger than the distance allowed by the user, then microorganisms are pushing each other function their mass and their distance (Eqs A26 – A31).

$$\vec{p} = \frac{kDist \cdot (r_m + r_{m+1}) - |\vec{v}|}{|\vec{v}|} \tag{A26}$$

$$a_m = 1 - \frac{X_m}{X_m + X_{m+1}} \; ; \; a_{m+1} = 1 - \frac{X_{m+1}}{X_m + X_{m+1}} \tag{A27}$$

$$x_{new,m} = x_{old} - (x_{m+1} - x_m) \cdot a_m \cdot \vec{p} \tag{A28}$$

$$y_{new,m} = y_{old} - (y_{m+1} - y_m) \cdot a_m \cdot \vec{p} \tag{A29}$$

$$x_{new,m+1} = x_{old} + (x_{m+1} - x_m) \cdot a_{m+1} \cdot \vec{p} \tag{A30}$$

$$y_{new,m+1} = y_{old} + (y_{m+1} - y_m) \cdot a_{m+1} \cdot \vec{p} \tag{A31}$$

3.1. Quadtree algorithm

Overlap detection and shoving computation between bacteria could be an expensive operation. Let a aggregate with 1000 bacteria. If we apply a rough overlap algorithm (comparing each pair of bacteria), would require $1\text{x}10^6$ operations (i.e., time complexity of $O(n^2)$). To improve shoving computation, we should reduce the number of checks that must be made. Two bacteria that are at opposite sites on aggregate (or far enough) cannot possibly overlap, so there is no need to check for an overlap between them. Here is where *quadtree* comes into play. Applying quadtree in overlap detection and shoving computation the number of checks is reduced significantly, turning the time complexity from $O(n^2)$ to $O(n \log n)$.

Quadtree is a tree data structure in which an internal node is sectioned in four child nodes, and each of those children could potentially be sectioned into four. Basically, quadtrees are used to split a 2D regional space by recursively subdividing it into four

regions. In this case, the number of bacteria in each subregion dictates if these regions must be divided or not – if a specific region holds a higher number of bacteria than the maximum number (so-called *capacity*), then this region will be divided into four new regions. Fig A4 shows an example of how quadtree works in a system in which it has a *capacity* of one bacterium (i.e., only one bacterium per region).

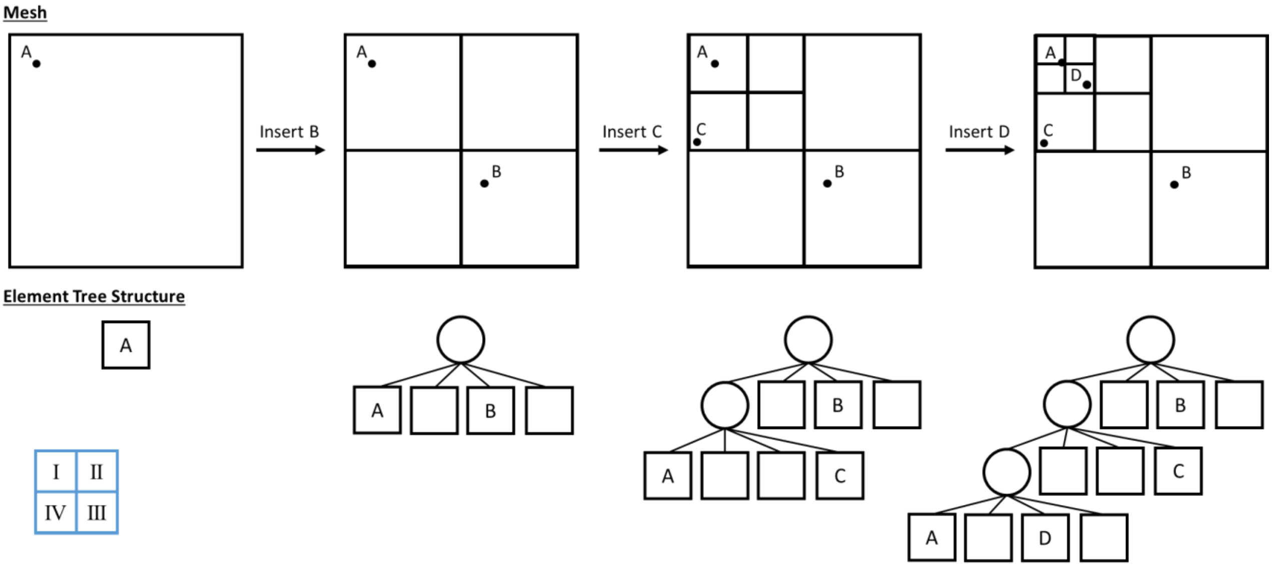

Fig A4. Quadtree representation (*capacity* = 1). Each circle represents a bacterium in the system. Subregions are labelled clockwise (see blue grid on bottom-left of figure).

Once understood how quadtree is created (mesh and data structure), it is time to figure out how quadtree plays in shoving algorithm. First, it is necessary to create a new quadtree mesh and tree data structure (Fig A5). Obviously, every time that a division occurs, quadtree must be updated for the new bacteria and their locations. Afterwards, each bacterium is *inserted* on quadtree, that is, it is set in a specific region (or node).

Before executing the overlap detection, the neighbours of all bacteria are established. For that, it is firstly created a *neighbourhood zone* that surrounds each bacterium (i.e., bacterium is on the centre of neighbourhood zone). As the quadtree is not updated after every shoving step, the *neighbourhood zone* is set to 4 times the maximum radius of bacteria. Then, for each bacterium, it is selected those regions (or subregions) which intersects with its *neighbourhood zone* and, subsequently, it is evaluated whether bacteria located in those intersect regions are within the neighbourhood zone or not (Fig A5).

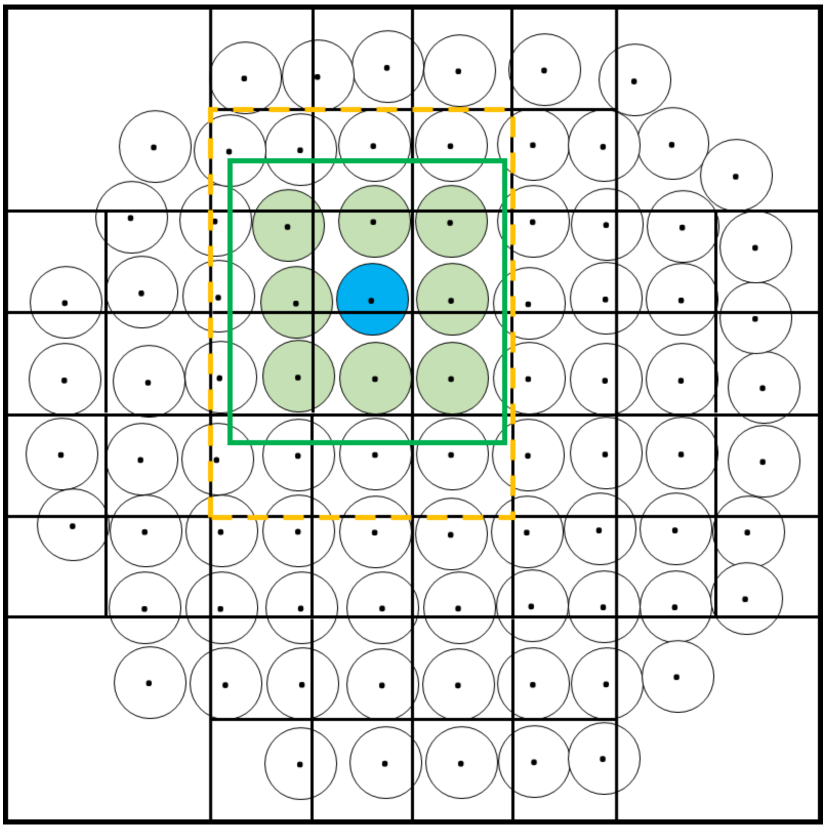

Fig A5. Selection of neighbours (green circles) of bacterium *m* (blue circle). The centre of circle (black dot) indicates the exact position of bacteria. In this example, quadtree *capacity* is 4 (i.e., maximum 4 bacteria per region). Green square represents the neighbourhood zone of bacterium *m* (blue circle), and dashed orange square shows all regions that intersect with the neighbourhood zone of bacterium *m*.

Finally, overlap assessment and shoving estimation (if needed) are only performed over the neighbours of bacteria (Eqs A24 – A31). In order to reduce the computational cost of shoving algorithm, *neighbourhood zone* of existing bacteria is not updated at any time, because bacteria will not move very far from original position. An overview of the shoving algorithm is presented in Fig A6.

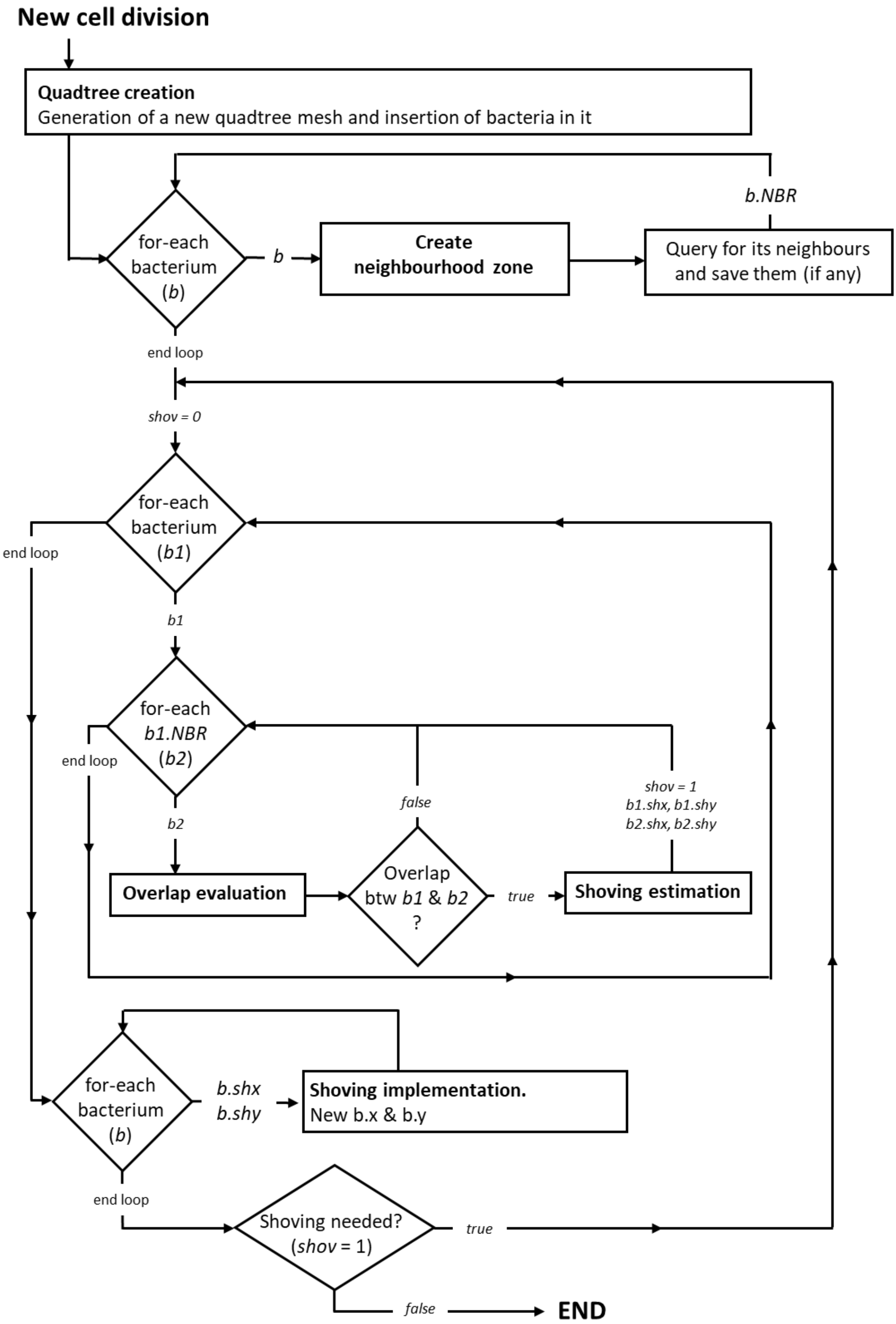

Fig A6. Scheme of shoving algorithm. Legend: *b.shx* and *b.shy* – displacement of bacterium *b* due to shoving of the others; *b.NBR* – neighbours of bacterium *b*.

4. Integration

An overall scheme of model integration and summary of model parameters are presented in Fig A7 and Table A1, respectively.

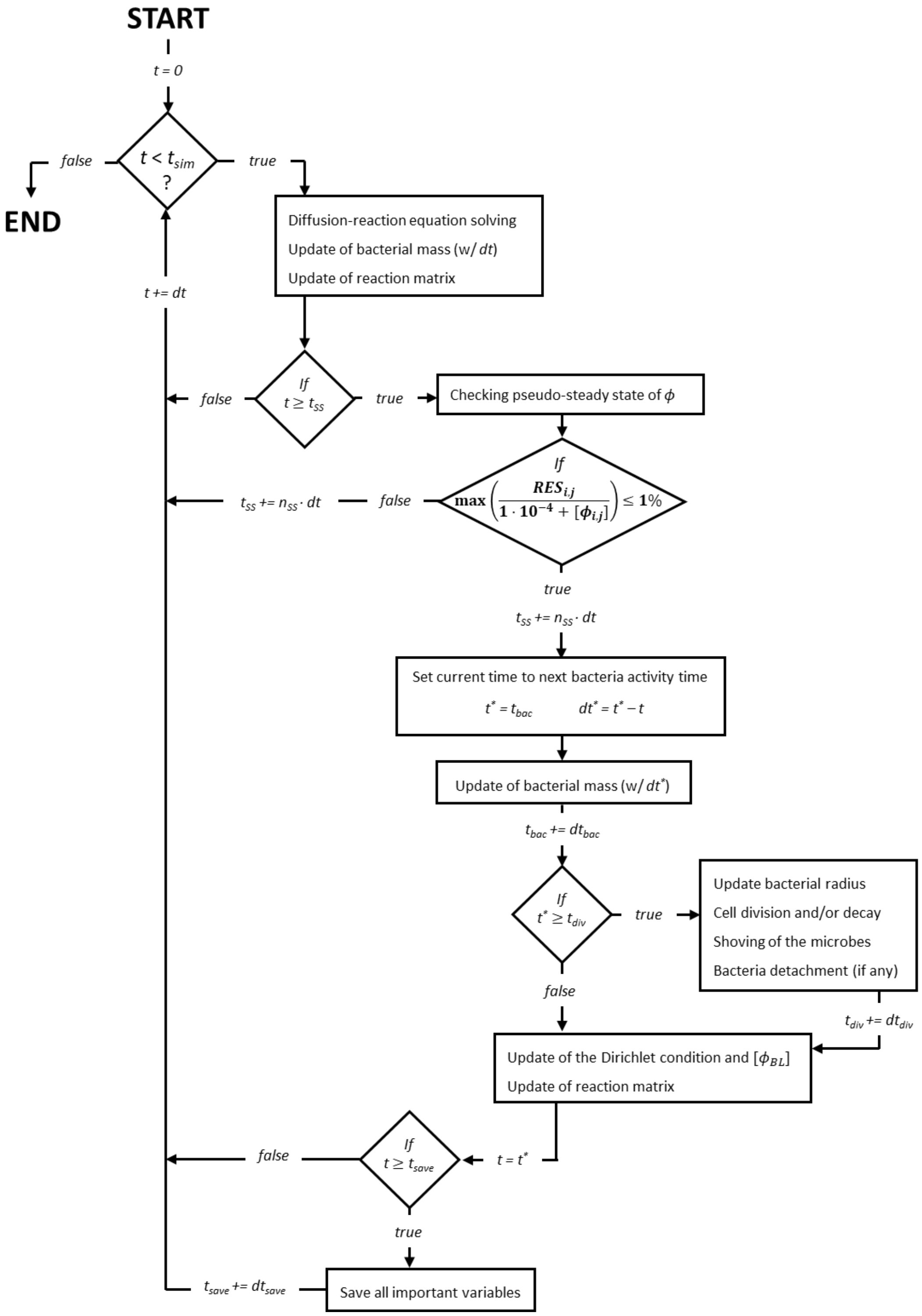

Fig A7. Algorithm scheme of the integration process.

Table A1. Summary of model parameters. Description and references of values used in the simulations of aggregate maturation.

	**Description**	**Value**	**Units**	**Ref.**
d_{max}	Maximum diameter of bacteria (*E. coli* longitude)	1.5	μm	[13]
ρ	Density of bacteria (considered constant)	1.1	$g \cdot cm^{-3}$	[14]
M_{max}	Maximum mass of bacteria (supposing a spherical shape)	2.09	pg	Est.[a]
M_{min}	Minimum mass of bacteria (10% of maximum mass)	0.21	pg	Est.
MW	Molecular weight of bacteria ($CH_{1.8}O_{0.5}N_{0.2}$)	24.6	$g \cdot mol^{-1}$	[15]
overlap	Maximum overlap allowed (10% of diameter of bacteria)	0.15	μm	–
Δx	Node length for coordinate x	2	μm	–
Δy	Node length for coordinate y	2	μm	–
N_x	Number of nodes for coordinate x	260	–	–
N_y	Number of nodes for coordinate y	260	–	–
Δt	Time step used to integrate diffusion-reaction equation	1×10^{-8}	h	–
Δt_{bac}	Time step used to check cell division	1.0	h	–
Δt_R	Time step used to integrate mass balances of reactor	1.0	h	–
blayer	Length of boundary layer	4	μm	–
D_A	Diffusion coefficient of substrate A in water (typical value)	3.60×10^{-6}	$m^2 \cdot h^{-1}$	–
D_B	Diffusion coefficient of substrate B in water (typical value)	3.60×10^{-6}	$m^2 \cdot h^{-1}$	–
D_C	Diffusion coefficient of substrate C in water (typical value)	3.60×10^{-6}	$m^2 \cdot h^{-1}$	–
D_{O2}	Diffusion coefficient of oxygen in water	7.56×10^{-6}	$m^2 \cdot h^{-1}$	[16]
tol	Tolerance relative of pseudo-steady state for any substrate	1.0	%	–
HRT	Initial hydraulic retention time of reactor	10.8	h	–

[a]Est. – Estimated

5. References

1. Mitri S, Xavier JB, Foster KR. Social evolution in multispecies biofilms. Proceedings of the National Academy of Sciences. 2011;108(supplement_2):10839-46. doi: doi:10.1073/pnas.1100292108.

2. Momeni B, Waite AJ, Shou W. Spatial self-organization favors heterotypic cooperation over cheating. Elife. 2013;2:e00960. Epub 2013/11/14. doi: 10.7554/eLife.00960. PubMed PMID: 24220506; PubMed Central PMCID: PMCPMC3823188.

3. Kehe J, Ortiz A, Kulesa A, Gore J, Blainey PC, Friedman J. Positive interactions are common among culturable bacteria. Science Advances. 2021;7(45):eabi7159. doi: doi:10.1126/sciadv.abi7159.

4. Palmer JD, Foster KR. Bacterial species rarely work together. Science. 2022;376(6593):581-2. doi: doi:10.1126/science.abn5093.

5. Thomas JW. Numerical Partial Differential Equations: Finite Difference Methods. Berlin, New York: Springer-Verlag1995. 437- p.

6. Kreft J-U, Booth G, Wimpenny JWT. BacSim, a simulator for individual-based modelling of bacterial colony growth. Microbiology. 1998;144(12):3275-87. doi: https://doi.org/10.1099/00221287-144-12-3275.

7. Kreft J-U, Picioreanu C, Wimpenny JWT, van Loosdrecht MCM. Individual-based modelling of biofilms. Microbiology. 2001;147(11):2897-912. doi: doi:10.1099/00221287-147-11-2897.

8. Briggs W, Henson V, McCormick S. A Multigrid Tutorial, 2nd Edition2000.

9. Pikulin VP, Pohozaev SI. Equations in Mathematical Physics: Birkhäuser Basel; 2001. 207 p.

10. Kleerebezem R, Van Loosdrecht MCM. A generalized method for thermodynamic state analysis of environmental systems. Critical Reviews in Environmental Science and Technology. 2010;40(1):1-54. doi: 10.1080/10643380802000974.

11. Heijnen JJ, van Dijken JP. In search of a thermodynamic description of biomass yields for chemotrophic growth of microorganisms. Biotechnology and Bioengineering. 1992;39(8):833-58. doi: 10.1002/bit.260390806.

12. Samet H, editor An Overview of Quadtrees, Octrees, and Related Hierarchical Data Structures1988; Berlin, Heidelberg: Springer Berlin Heidelberg.

13. Reshes G, Vanounou S, Fishov I, Feingold M. Cell shape dynamics in Escherichia coli. Biophys J. 2008;94(1):251-64. Epub 2007/08/31. doi: 10.1529/biophysj.107.104398. PubMed PMID: 17766333.

14. Loferer-Krößbacher M, Klima J, Psenner R. Determination of Bacterial Cell Dry Mass by Transmission Electron Microscopy and Densitometric Image Analysis.

Applied and Environmental Microbiology. 1998;64(2):688-94. doi: 10.1128/aem.64.2.688-694.1998.

15. Grosz R, Stephanopoulos G. Statistical mechanical estimation of the free energy of formation of E. coli biomass for use with macroscopic bioreactor balances. Biotechnol Bioeng. 1983;25(9):2149-63. Epub 1983/09/01. doi: 10.1002/bit.260250904. PubMed PMID: 18574813.

16. Ferrell RT, Himmelblau DM. Diffusion coefficients of nitrogen and oxygen in water. Journal of Chemical & Engineering Data. 1967;12(1):111-5. doi: 10.1021/je60032a036.
