# Supplementary material for: Environmental and ecological controls of the spatial distribution of microbial populations in aggregates
Source: PLoS Comput Biol. 2022 Dec 19;18(12):e1010807. doi: 10.1371/journal.pcbi.1010807 (PMC9810174; doi:10.1371/journal.pcbi.1010807)
Supplement: S1 Table — (PDF) [file pcbi.1010807.s002.pdf]

S1 Table. Kinetics of all simulation setups

<table>
<tr><th>Process</th><th>Process rate (h^{-1})</th><th>Kinetic parameters</th></tr>
<tr><td colspan="2">Neutralism</td><td rowspan="20">Maximum growth rate (μ_{max}):
$\mu_{max,B1} = 1d^{-1}$
$\mu_{max,B2} = 1d^{-1}$
$\mu_{max,B3} = 1d^{-1}$
Decay coefficient[a] (b):
$b_{B1} = 0.25d^{-1}$
$b_{B2} = 0.25d^{-1}$
$b_{B3} = 0.25d^{-1}$
Affinity constant for A (K_A):
$K_{A,B1} = 0.01mM$
$K_{A,B2} = 0.01mM$
$K_{A,B3} = 0.01mM$
Affinity constant for B (K_B):
$K_{B,B1} = 0.01mM$
$K_{B,B2} = 0.01mM$
$K_{B,B3} = 0.01mM$
Affinity constant for C (K_C):
$K_{C,B1} = 0.01mM$
$K_{C,B2} = 0.01mM$
$K_{C,B3} = 0.01mM$
Affinity constant for O_2 (K_{O2}):
$K_{O_2,B1} = 0.001mM$
$K_{O_2,B2} = 0.001mM$
$K_{O_2,B3} = 0.001mM$</td></tr>
<tr><td>Growth of B1</td><td>$\mu_{max,B1} \cdot \frac{[A]}{K_{A,B1}+[A]} \cdot X_{B1}$</td></tr>
<tr><td>Growth of B2</td><td>$\mu_{max,B2} \cdot \frac{[B]}{K_{B,B2}+[B]} \cdot X_{B2}$</td></tr>
<tr><td>Growth of B3</td><td>$\mu_{max,B3} \cdot \frac{[C]}{K_{C,B3}+[C]} \cdot X_{B3}$</td></tr>
<tr><td colspan="2">Competition</td></tr>
<tr><td>Growth of B1</td><td>$\mu_{max,B1} \cdot \frac{[A]}{K_{A,B1}+[A]} \cdot X_{B1}$</td></tr>
<tr><td>Growth of B2</td><td>$\mu_{max,B2} \cdot \frac{[A]}{K_{A,B2}+[A]} \cdot X_{B2}$</td></tr>
<tr><td>Growth of B3</td><td>$\mu_{max,B3} \cdot \frac{[A]}{K_{A,B3}+[A]} \cdot X_{B3}$</td></tr>
<tr><td colspan="2">Commensalism</td></tr>
<tr><td>Growth of B1</td><td>$\mu_{max,B1} \cdot \frac{[A]}{K_{A,B1}+[A]} \cdot X_{B1}$</td></tr>
<tr><td>Growth of B2</td><td>$\mu_{max,B2} \cdot \frac{[B]}{K_{B,B2}+[B]} \cdot X_{B2}$</td></tr>
<tr><td>Growth of B3</td><td>$\mu_{max,B3} \cdot \frac{[C]}{K_{C,B3}+[C]} \cdot X_{B3}$</td></tr>
<tr><td colspan="2">Competition + Commensalism</td></tr>
<tr><td>Growth of B1</td><td>$\mu_{max,B1} \cdot \frac{[A]}{K_{A,B1}+[A]} \cdot \frac{[O_2]}{K_{O2,B1}+[O_2]} \cdot X_{B1}$</td></tr>
<tr><td>Growth of B2</td><td>$\mu_{max,B2} \cdot \frac{[B]}{K_{B,B2}+[B]} \cdot \frac{[O_2]}{K_{O2,B2}+[O_2]} \cdot X_{B2}$</td></tr>
<tr><td>Growth of B3</td><td>$\mu_{max,B3} \cdot \frac{[C]}{K_{C,B3}+[C]} \cdot \frac{[O_2]}{K_{O2,B3}+[O_2]} \cdot X_{B3}$</td></tr>
<tr><td colspan="2">For all simulation setups</td></tr>
<tr><td>Decay of B1</td><td>$b_{B1} \cdot X_{B1}$</td></tr>
<tr><td>Decay of B2</td><td>$b_{B2} \cdot X_{B2}$</td></tr>
<tr><td>Decay of B3</td><td>$b_{B3} \cdot X_{B3}$</td></tr>
</table>

[a]Decay ratio is 25% of maximum growth rate (μ_{max}) [1].

References

1. Bodegom PV. Microbial maintenance: a critical review on its quantification. Microbial Ecology. 2007;53:513-23. doi: 10.1007/s00248-006-9049-5.
