# Supplementary material for: Environmental and ecological controls of the spatial distribution of microbial populations in aggregates
Source: PLoS Comput Biol. 2022 Dec 19;18(12):e1010807. doi: 10.1371/journal.pcbi.1010807 (PMC9810174; doi:10.1371/journal.pcbi.1010807)
Supplement: S2 Table — (PDF) [file pcbi.1010807.s003.pdf]

S2 Table. Stoichiometries of all simulation setups

Process	Compounds[a]							
	A	B	C	D	O_2	B1	B2	B3
**Neutralism (A,B,C)**								
Growth of B1	$-1/Y_{B1}$[b]	0	0	$1/Y_{B1}$	0	1	0	0
Growth of B2	0	$-1/Y_{B2}$[b]	0	$1/Y_{B2}$	0	0	1	0
Growth of B3	0	0	$-1/Y_{B3}$[b]	$1/Y_{B3}$	0	0	0	1
**Competition (A)**								
Growth of B1	$-1/Y_{B1}$	$1/Y_{B1}$	0	0	0	1	0	0
Growth of B2	$-1/Y_{B2}$	$1/Y_{B2}$	0	0	0	0	1	0
Growth of B3	$-1/Y_{B3}$	$1/Y_{B3}$	0	0	0	0	0	1
**Commensalism (A,B,C)**								
Growth of B1	$-1/Y_{B1}$	$1/Y_{B1}$	0	0	0	1	0	0
Growth of B2	0	$-1/Y_{B2}$	$1/Y_{B2}$	0	0	0	1	0
Growth of B3	0	0	$-1/Y_{B3}$	$1/Y_{B3}$	0	0	0	1
**Competiton (O_2) + Commensalism (A,B,C)**								
Growth of B1	$-1/Y_{B1}$	$1/Y_{B1}$	0	0	$-1/Y_{B1}$	1	0	0
Growth of B2	0	$-1/Y_{B2}$	$1/Y_{B2}$	0	$-1/Y_{B2}$	0	1	0
Growth of B3	0	0	$-1/Y_{B3}$	$1/Y_{B3}$	$-1/Y_{B3}$	0	0	1
**For all simulation setups**								
Decay of B1	0	0	0	0	0	-1	0	0
Decay of B2	0	0	0	0	0	0	-1	0
Decay of B3	0	0	0	0	0	0	0	-1

[a]Units: $mol \cdot L^{-1}$

[b]All bacteria have the same growth yield: $Y_{B1} = Y_{B2} = Y_{B3} = 0.01$ (mol X)·(mol S)$^{-1}$
