# Supplementary material for: Environmental and ecological controls of the spatial distribution of microbial populations in aggregates
Source: PLoS Comput Biol. 2022 Dec 19;18(12):e1010807. doi: 10.1371/journal.pcbi.1010807 (PMC9810174; doi:10.1371/journal.pcbi.1010807)
Supplement: S3 Table — For each setup, eco-interactions between microbial species, environment of the reactor, and substrate concentrations on bulk liquid ([A], [B], [C] and [D]) are specified. Also, it includes in which Figures the results are shown. (PDF) [file pcbi.1010807.s004.pdf]

S3 Table. Details of simulation experiments

Setup	Specifications	Figures
**Setup 1: Saturation**<br>One eco-interaction	· Replicates (3-6) of each simulation experiment<br>· Substrate concentrations (A, B and C): $[S]_T$: 100mM, 10mM, 1mM [a]<br>· Ecological interactions and influent characterization:<br>Neutralism: feeding of A, B, and C → $[A] = [B] = [C] = [S]_T/3$<br>Competition: feeding of A → $[A] = [S]_T$<br>Commensalism: feeding of A → $[A] = [S]_T$	All eco-inter.<br>Fig 2<br>S1 and S2 Figs<br>S1 and S2 Videos
**Setup 2: Anaerobic**<br>One eco-interaction	· Replicates (3-6) of each simulation experiment<br>· Substrate concentrations (A, B and C): $[S]_T$: 1mM, 0.5mM, 0.2mM, 0.1mM<br>· Ecological interactions and influent characterization:<br>Neutralism: feeding of A, B, and C → $[A] = [B] = [C] = [S]_T/3$<br>Competition: feeding of A → $[A] = [S]_T$<br>Commensalism: feeding of A → $[A] = [S]_T$	Neutralism<br>Fig 3<br>Competition<br>Figs 4 and 8<br>S3 Video<br>Commensalism<br>Fig 5<br>S4 Fig<br>All eco-inter.<br>S3 Fig
**Setup 3: Aerobic**<br>Two eco-interactions	· Replicates (3-6) of each simulation experiment<br>· Substrate concentrations (A, B and C): $[S]_T$: 1mM, 0.5mM, 0.1mM, 0.05mM<br>· Oxygen concentrations: $[O_2]$: 10mg·L^{-1}, 6mg·L^{-1}, 3.75mg·L^{-1}, 1.90mg·L^{-1}, 1mg·L^{-1}, 0.5mg·L^{-1}<br>· Ecological interactions and bulk liquid characterization:<br>Commensalism + competition: feeding of A and O_2 → $[A]_{BL} = [S]_T$	Commensalism plus competition<br>Figs 6, 7 and 8<br>S5 – S10 Figs<br>S3 Video

[a] Consideration until substrates started to become limiting inside aggregate.
