# Supplementary figures and images for: Environmental and ecological controls of the spatial distribution of microbial populations in aggregates
Source: PLoS Comput Biol. 2022 Dec 19;18(12):e1010807. doi: 10.1371/journal.pcbi.1010807 (PMC9810174; doi:10.1371/journal.pcbi.1010807)

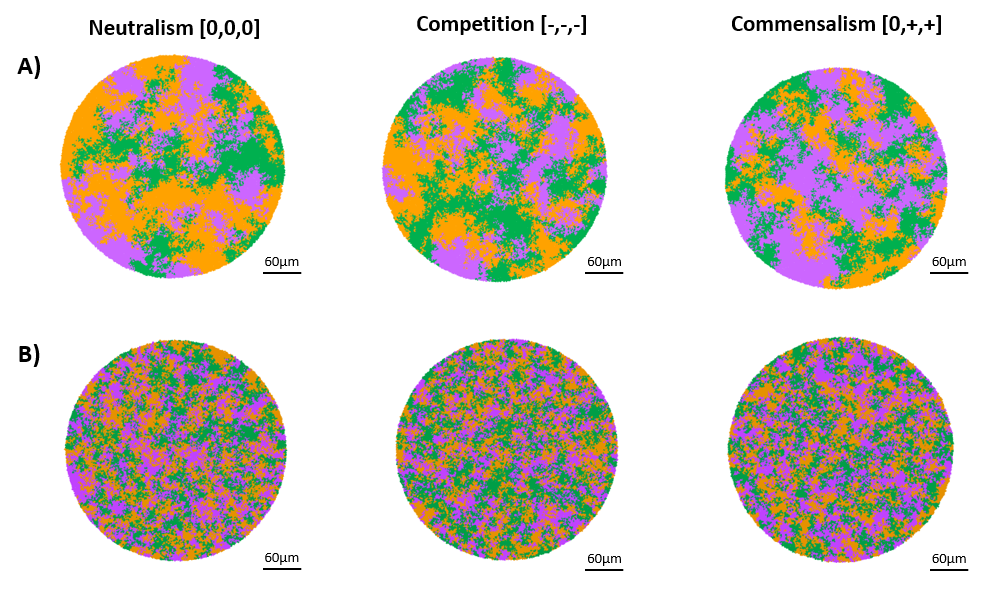

Supplement: S1 Fig — (A) Aggregate pictures captured at 8 d of simulation starting with an inoculum size of 20 μm (diameter) and considering diffusion resistance of substrates. (B) Aggregate pictures captured at 4 d of simulation starting with an inoculum size of 160 μm (replicating the starting point of Mitri et al. (2016)) [29] and removing the substrate gradients (no diffusion resistance). None of the simulations are in steady state yet. (TIF) [file pcbi.1010807.s005.tif]

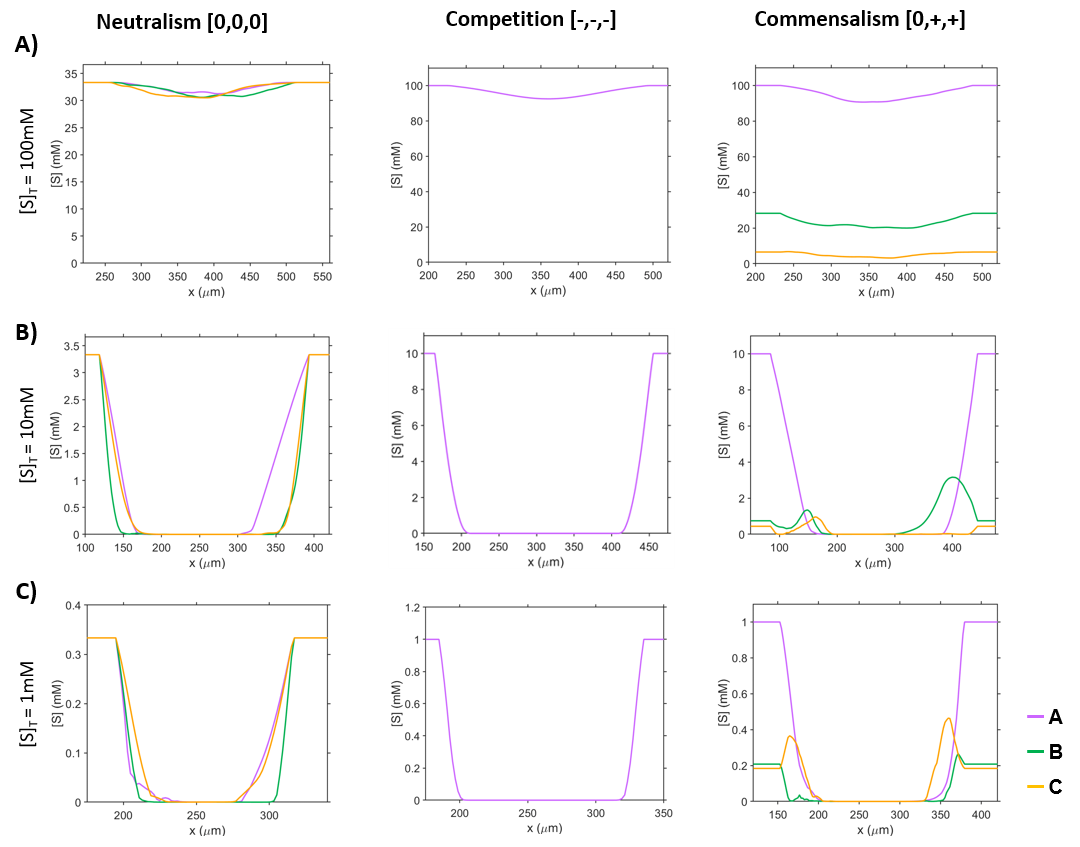

Supplement: S2 Fig — (A) Substrate profiles from simulations at [S]T = 100 mM (t = 8 d). (B) Substrate profiles from simulations at [S]T = 10 mM (t = 10 d). (C) Substrate profiles from simulations at [S]T = 1 mM (t = 15d). Legend: [A]–purple line; [B]–green line; [C]–orange line. (TIF) [file pcbi.1010807.s006.tif]

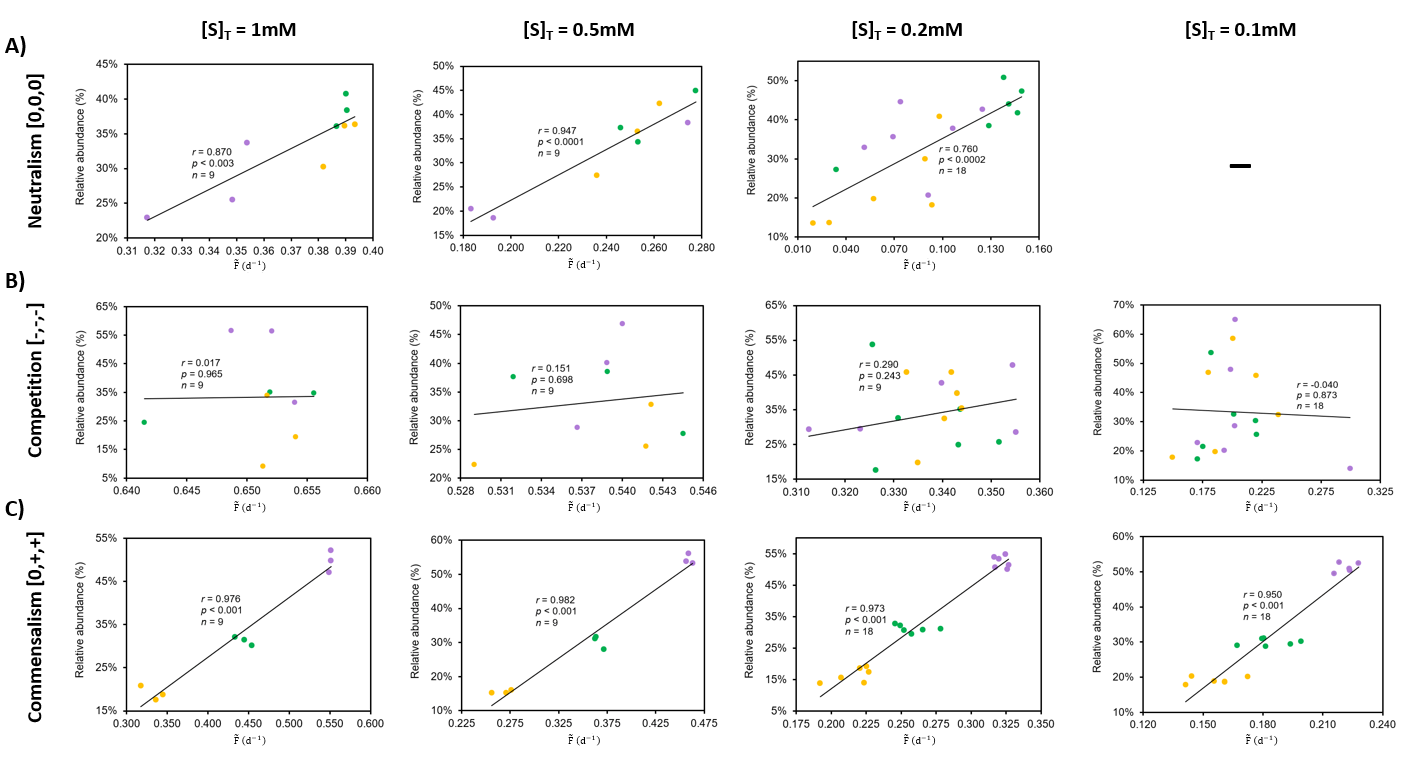

Supplement: S3 Fig — (A) Simulations considering neutralism. (B) Simulations considering competition. (C) Simulations considering commensalism. Legend: B1– purple circles; B2 –green circles; B3 –orange circles. (TIF) [file pcbi.1010807.s007.tif]

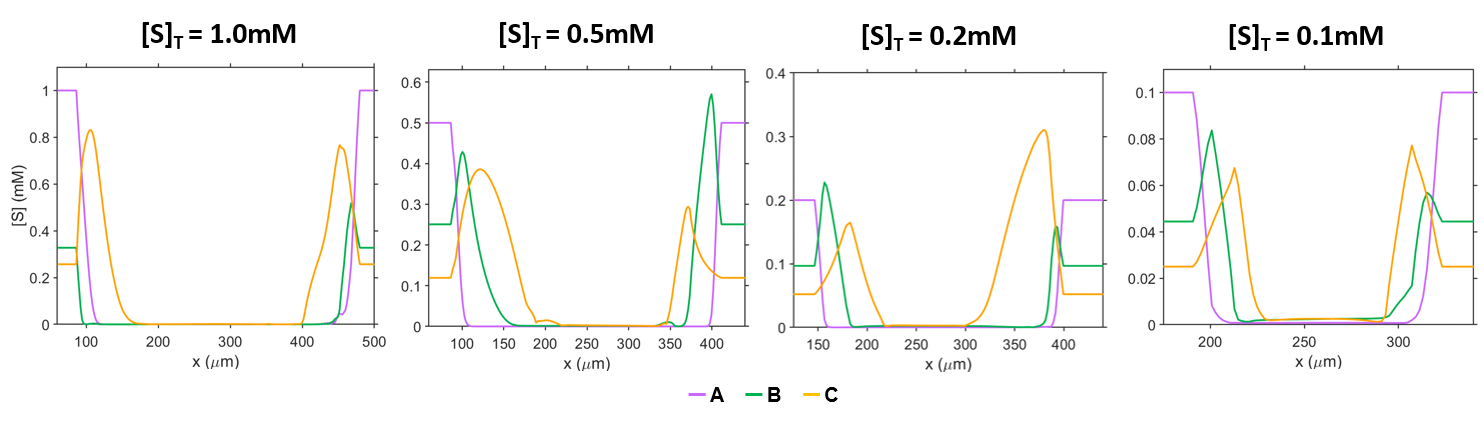

Supplement: S4 Fig — Legend: [A]–purple line; [B]–green line; [C]–orange line. (TIF) [file pcbi.1010807.s008.tif]

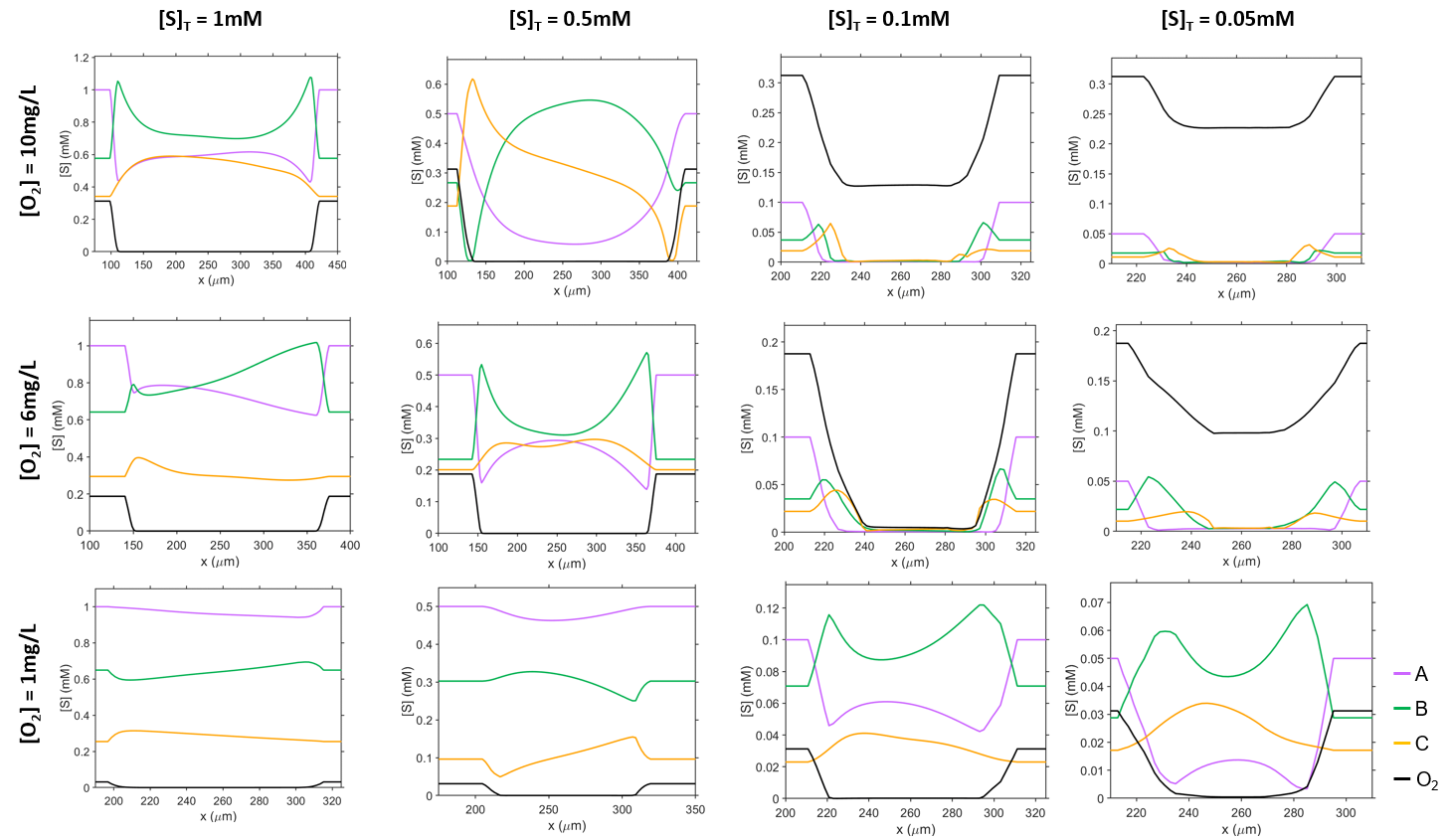

Supplement: S5 Fig — Legend: [A]–purple line; [B]–green line; [C]–orange line; [O2]–black line. (TIF) [file pcbi.1010807.s009.tif]

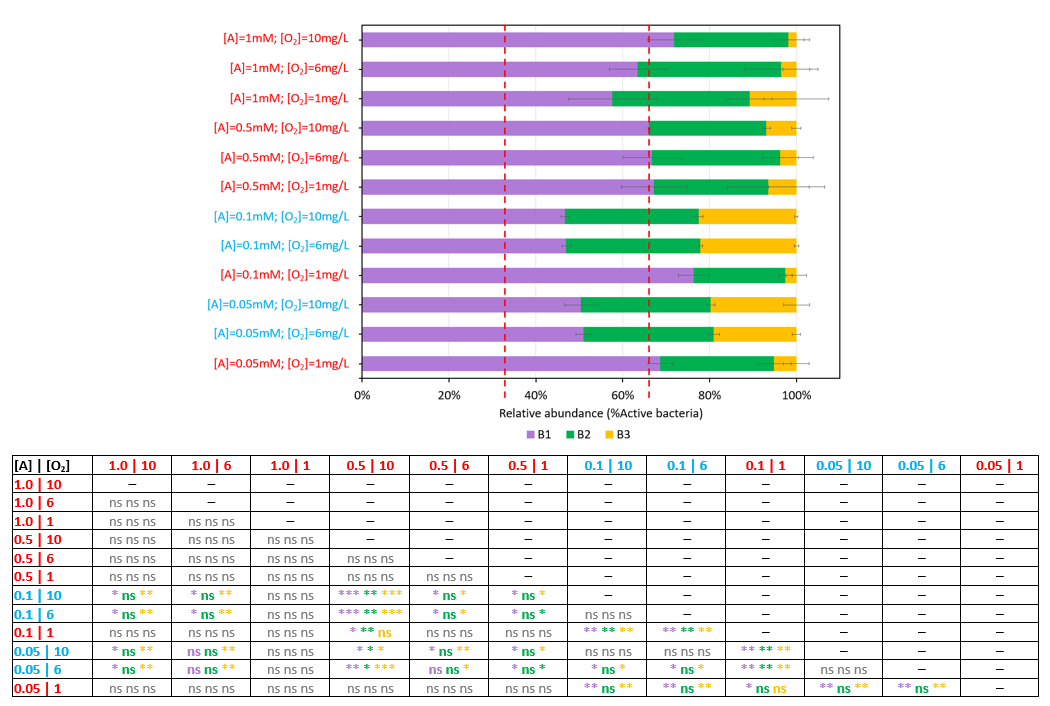

Supplement: S6 Fig — Dashed red lines indicate 33.33% and 66.66% relative abundances. Colour of asterisks points out what bacteria it refers to. In the table are shown the significant level of the difference between B1, B2 and B3 relative abundances. Significance level legend: ns, not significant; *, p < 0.05; **, p < 0.01; ***, p < 0.001. Colours of y-axis text and table headers indicate the ecological environment (and spatial distribution of microbial populations) of simulation experiments: red–competitive environment (columned stratification); blue–commensal environment (layered stratification). (TIF) [file pcbi.1010807.s010.tif]

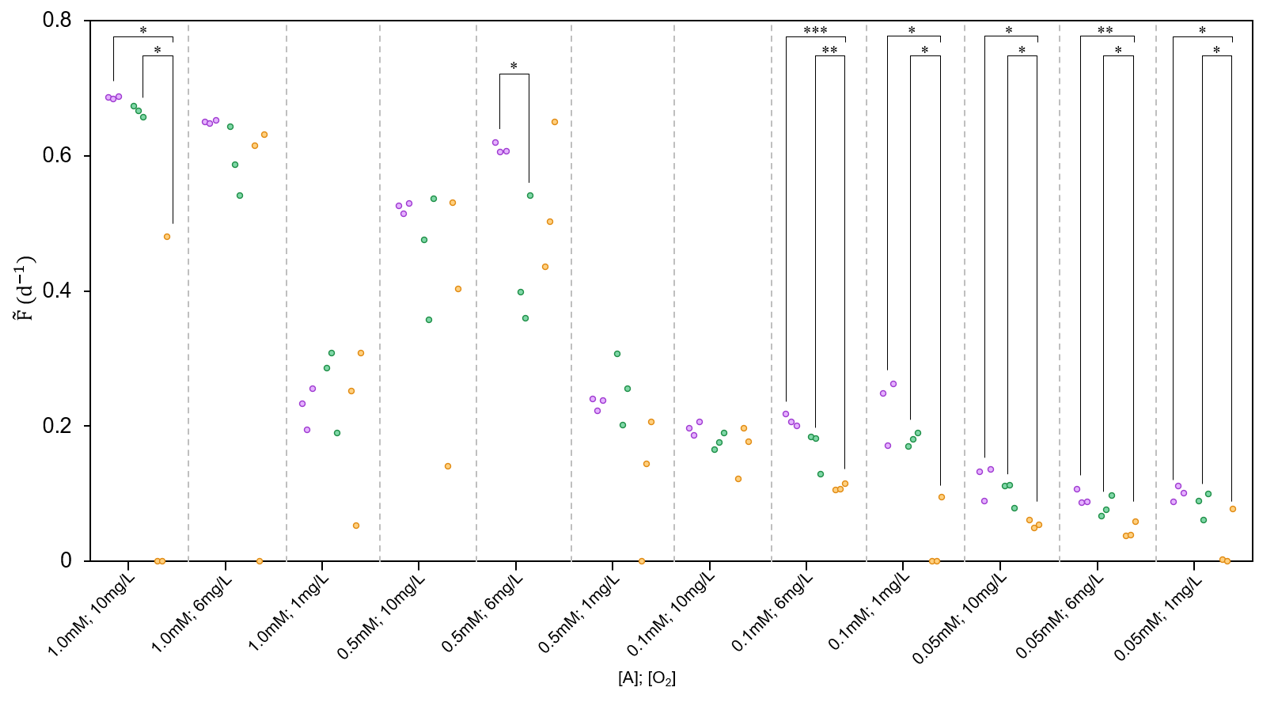

Supplement: S7 Fig — Asterisks indicate the significance level of the difference between B1, B2 and B3 specific growth rate. Significance level legend: ns, not significant; *, p < 0.05; **, p < 0.01; ***, p < 0.001. (TIF) [file pcbi.1010807.s011.tif]

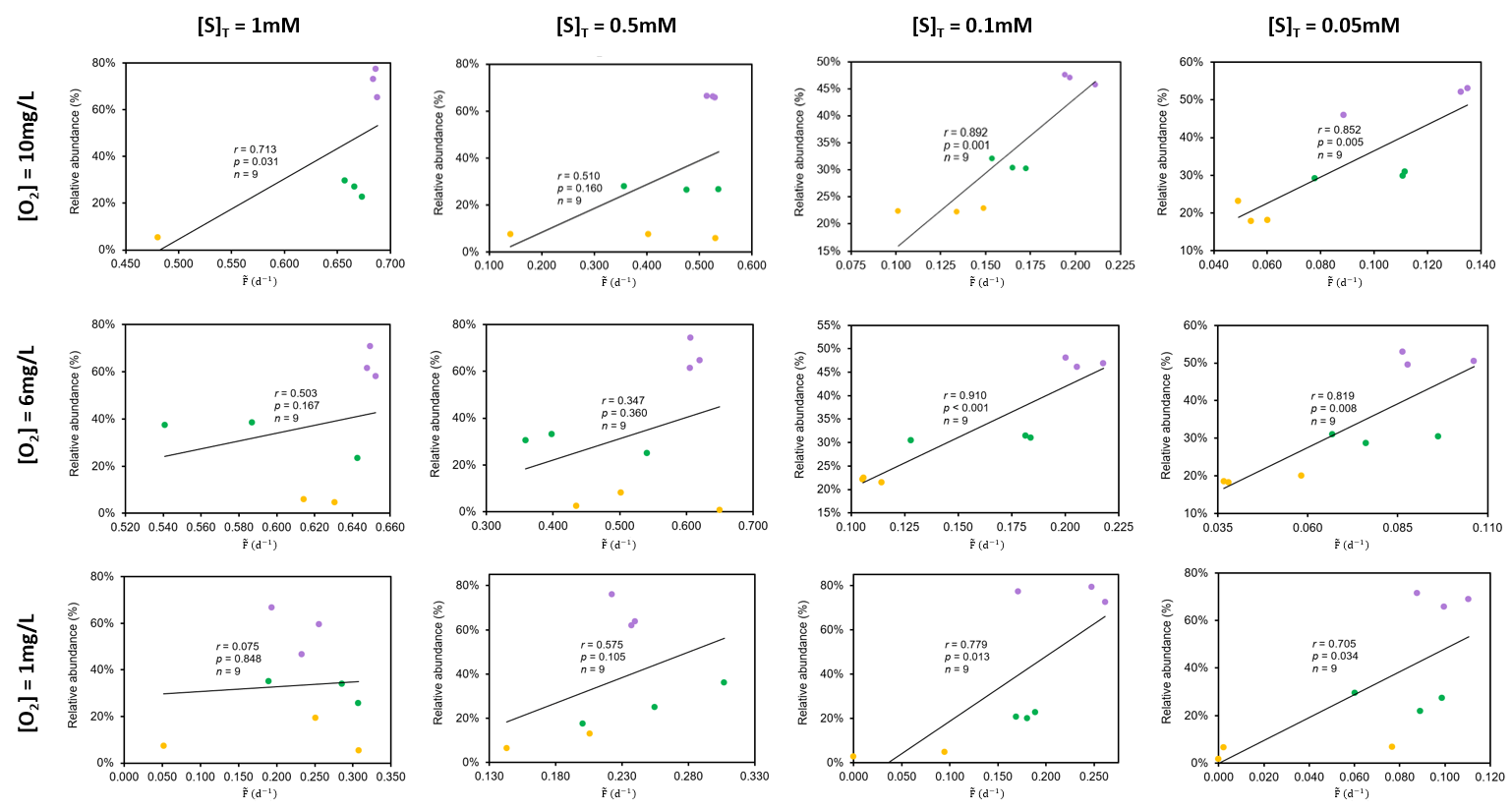

Supplement: S8 Fig — Legend: B1– purple circles; B2 –green circles; B3 –orange circles. (TIF) [file pcbi.1010807.s012.tif]

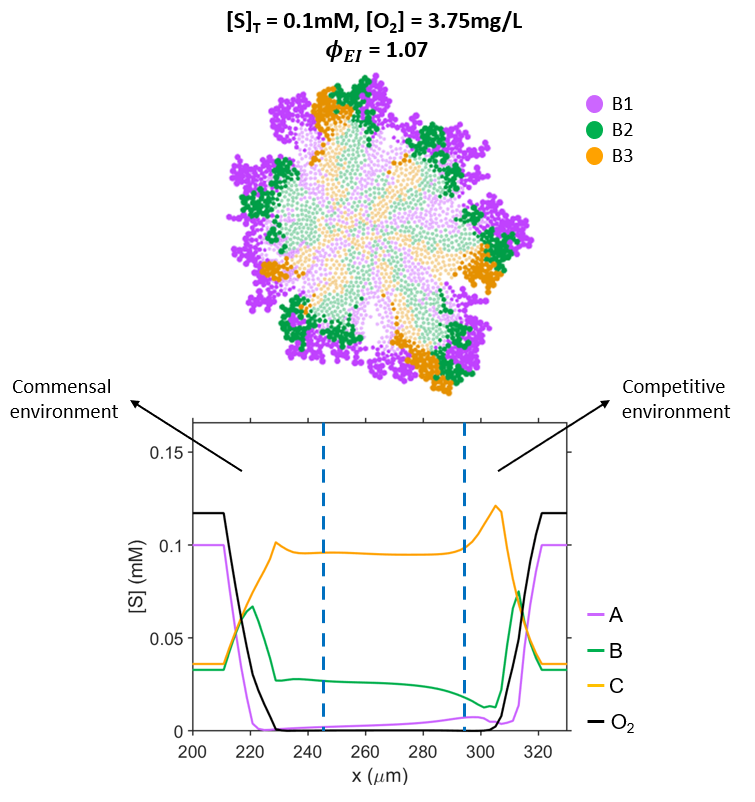

Supplement: S9 Fig — Inactive bacteria are shown in a lighter colour. The substrate profiles are from the transverse plane of aggregate. (TIF) [file pcbi.1010807.s013.tif]

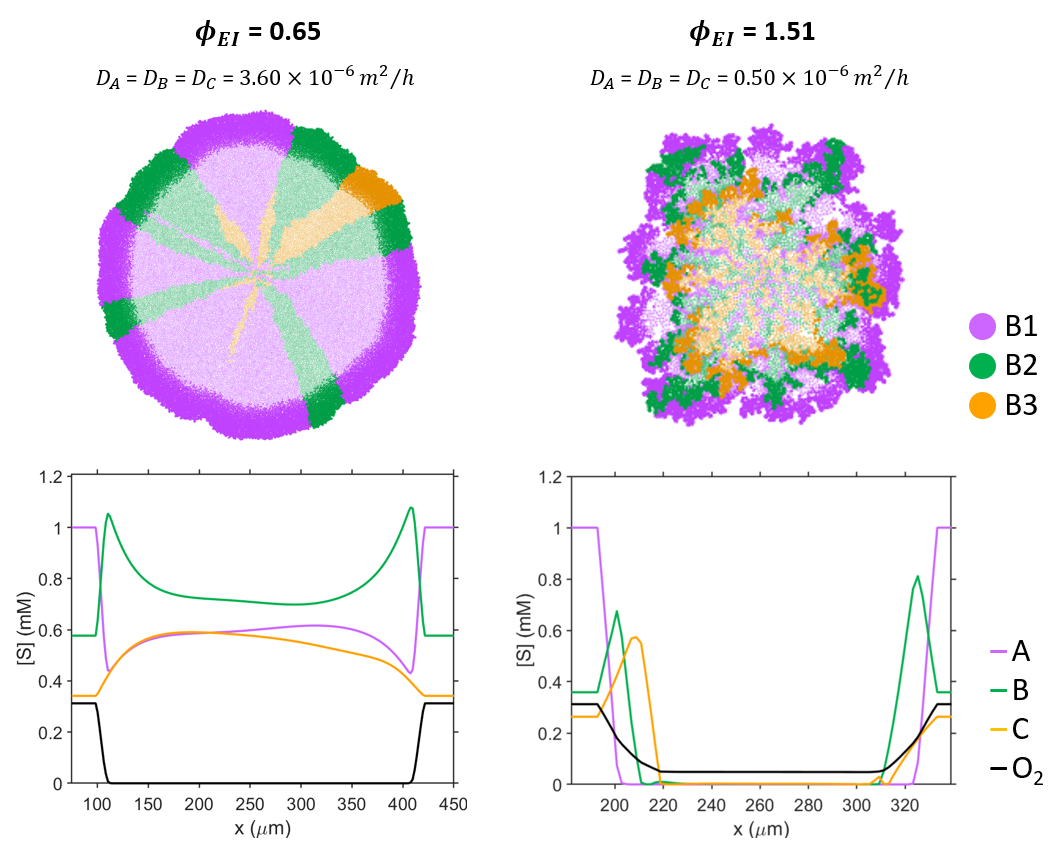

Supplement: S10 Fig — Inactive bacteria are shown in a lighter colour. The substrate profiles (S11 Fig) are from the transverse plane of aggregates. Diffusion coefficient (Di in Eq 1) states the diffusion rate of certain substance into the fluid. Therefore, those substrates with lower diffusion coefficient will tend to be the limiting substrate in the aggregate, establishing the ecological environment and, consequently, the spatial distribution of microbial populations. In order to illustrate the influence of diffusion constant, we simulated again one of the cases of concurrence commensalism (substrate A) and competition (oxygen) but now reducing the diffusion coefficient of substrates A, B, C and D (from 3.60x10^-6^ m2/h to 0.5x10^-6^ m2/h). As example, the environment with 1.0 mM of A and 10.0 mg/L of O2 (competitive environment, ϕEI = 0.65) was applied starting with the same inoculum. With the new diffusion coefficients, substrate A (instead of O2) was the most limiting, obtaining a layered stratification of microbial populations (commensal environment, ϕEI = 1.51). (TIF) [file pcbi.1010807.s014.tif]

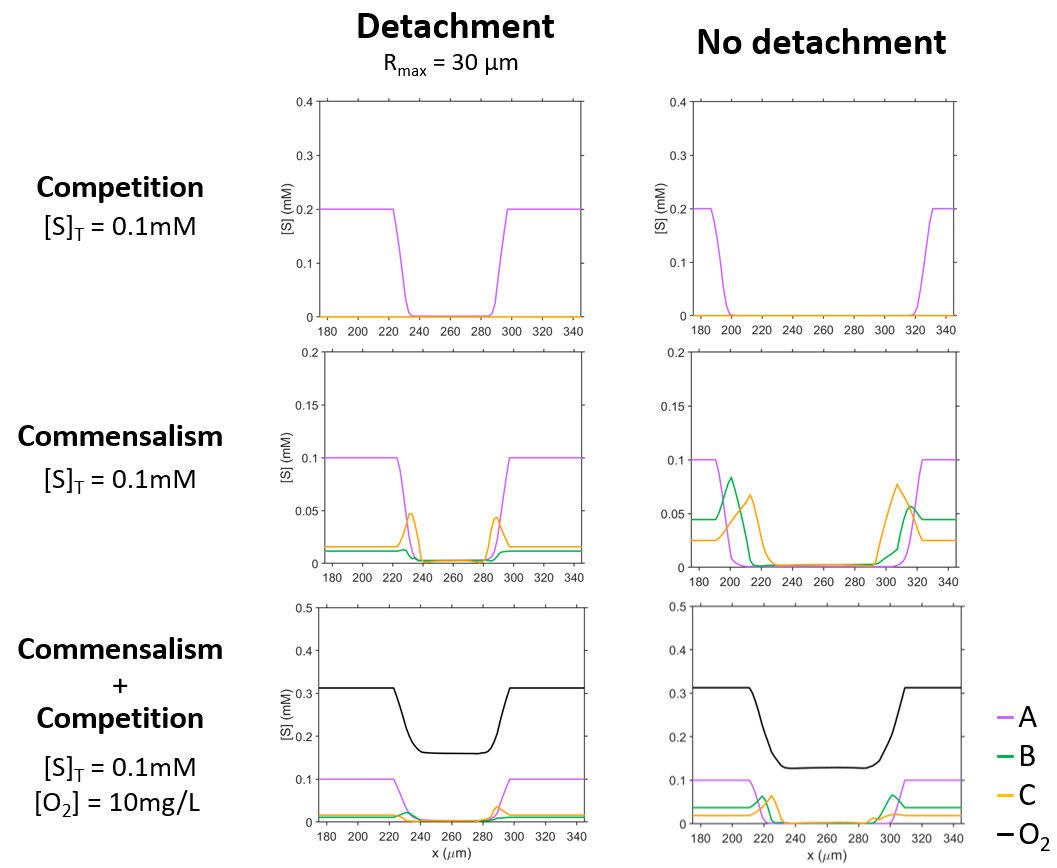

Supplement: S11 Fig — (TIF) [file pcbi.1010807.s015.tif]
