# Supplementary material for: Environmental and ecological controls of the spatial distribution of microbial populations in aggregates
Source: PLoS Comput Biol. 2022 Dec 19;18(12):e1010807. doi: 10.1371/journal.pcbi.1010807 (PMC9810174; doi:10.1371/journal.pcbi.1010807)
Supplement: Attachment — Submitted filename: Peer review.pdf [file pcbi.1010807.s019.pdf]

Review of Manuscript Number PCOMPBIOL-D-22-01033
"Environmental and ecological controls of the spatial distribution of microbial populations in aggregates"

The authors present a model of ecological interactions in bacterial aggregates. Using individual-based computational modelling they represent several different interactions and substrates. They show how these function in isolation and in interaction to form different spatial patterns, depending on the interactions and the limiting substrate. The introduction of the 'Eco-interaction modulus' is particularly interesting, with which they can describe what environmental conditions lead to which ecological interactions becoming dominant.

The paper is well-structured and the simulations are generally clearly represented. The visualisations are well-made and attractive, and the videos are a useful supplement. However, improvements are necessary, particularly to place the study in its biological context, to emphasise the new insights gained, and in the handling of statistics.

Major remarks:

1. Radial and concentric organisation of microbial populations depending on ecological interactions, and the concept that ecological relationships and the environment interact to shape the ecosystem, are not novel findings. These are already modelled in detail in, for example, reference [20] (Momeni et al.). The paper needs more comparison with existing models, such as [19] (Xavier et al) and [20] (Momeni et al). In particular, the authors should investigate what distinguishes their model from these previous models, and what new insights they have gained.
2. There should also be additional comparison in the discussion of the new insights with the available literature on *in vitro* and *in vivo* biological aggregate communities. Which of the observed patterns have previously been observed *in vivo* or *in vitro*? Is there an unexplained pattern that this model can explain?
3. It is unclear how the visualisations or statistics are performed with respect to the replicates in, for example, Fig. 3B and fig 4D. Are all fitness measurements from each replicate combined for each species? This seems to me to violate the assumption of independent observations. Multiple fitness values derived from the same replicate cannot be considered independent observations. This should either be corrected, or the reasoning should be described.
4. A possibly related question: How do you explain the very strongly significant differences between some species in the neutral condition of Fig. 3B? It seems to me that any differences between species in the neutralism condition would be the result of stochastic variation, and should not be so strongly significant. This may be the result of treating related observations as independent. In addition, if the variation between replicates is high, more than three replicates should be performed per condition.
5. There are references to an implementation of the "protection" eco-interaction in the text, but no results outside of the single run of figure S8. This condition should either be expanded upon, or be removed from the text if it is not analysed in any depth.

Minor remarks:

1. It is unclear at what point in the simulation measurements were taken in Fig. 3C. At line 108 you state that aggregates grow until steady state is reached, but you do not state when this is reached in Fig. 3, or if this is reached in Fig. 2. If both are in steady

state, why is the shape of the colony for the 1mM condition of Fig. 3C very different from that in Fig. 2C for the same condition?.

2. The description of the model at line 98 is insufficient to understand the subsequent results without also reading the methods - particularly the ‘colony size’ referred to frequently should be defined around line 98.
3. What is colony size relative to in Fig. 4D?
4. A link to the code is included, but the readme is so brief that it remains unclear to me how I should run the code, as someone not very familiar with matlab.
5. Line 260 - You introduce competition for O2 here as if that was already a factor, but you have not mentioned it previously.
6. How exactly do you determine when/if steady state has been reached? Would the results be greatly altered by placing it elsewhere? What is the variability between runs and conditions in reaching this steady state?
7. The paper is generally well-structured, but needs additional editing for grammar and spelling throughout the paper. Some examples:

- Line 37 revel -> reveal
- Line 38 dictates -> dictate
- Line 59 are -> is
- Line 69 oxidizes -> oxidizers
- Line 70 eco-interaction ->eco-interactions
- Line 89-91: I do not understand the use of “would” here - the model indicates this process already occurs
- Line 118 - You mean produced here, not metabolised
- Fig. 1 Ammensalism -> amensalism
