# Supplementary material for: Environmental and ecological controls of the spatial distribution of microbial populations in aggregates
Source: PLoS Comput Biol. 2022 Dec 19;18(12):e1010807. doi: 10.1371/journal.pcbi.1010807 (PMC9810174; doi:10.1371/journal.pcbi.1010807)
Supplement: Attachment — Submitted filename: Letter-referees_EMartinez-Rabertetal2022.docx [file pcbi.1010807.s020.docx]

Glasgow, 31^st^ October 2022

Dear Editors,

Please find enclose the detailed answers to the comments that referees have made, and the revised version of the manuscript “*Environmental and ecological controls of the spatial distribution of microbial populations in aggregates*” by Eloi Martinez-Rabert, Chiel van Amstel, Cindy Smith, William T. Sloan and Rebeca Gonzalez-Cabaleiro for publication in *PLOS Computational Biology*. As indicated, two versions of the revised manuscript have been submitted: one with all revised portions of the manuscript highlighted in red font; and other without highlighting the modifications (clean version).

We believe we have been able to address all the reviewer comments positively and that we have modified the manuscript substantially addressing all the concerns reviewers had. We like to thank all the reviewers for their valuable inputs that have helped us to improve our manuscript substantially.

We hope the manuscript can now be accepted for publication in *PLOS Computational Biology*.

Yours sincerely,

MSc Eloi Martinez-Rabert

Advanced Research Centre (ARC),

Chapel Lane, Glasgow, G11 6EW

*2424069M@student.gla.ac.uk*

**Answers to the Reviewer of the Manuscript:**

**“Environmental and ecological controls of the spatial distribution of microbial populations in aggregates”**

We appreciate the comments of the reviewers and believe their suggestion have allowed us to improve the quality of our manuscript. Please find our replies to the reviewer’s comments in the following section.

**Reviewer #1:**

**Major points:**

1. I would encourage the authors to better explain the difference between the kinetic growth parameters (assumed to be identical) and the stochiometric constrains (assumed to be variable). At multiple places in the text, I got confused at to what exactly was identical, and what was different. I had to go back to the supplement multiple times, and that is not ideal for your readers. A suggestion would be to design a little cartoon of the stoichiometry to complement the text-based descriptions (e.g. [0,+,+]), which you can then add above each column in the figures so that the readers immediately know what that implies.

**Answer**

In the main text Table 1 has been added to clarify the metabolic stoichiometries used. Additionally, text modifications have been added to better explain the ecological implications of each of the stoichiometries selected.

I also find it surprising that these three main interaction regimes that are studied are not mentioned in the abstract specifically.

**Answer**

We think this is a good point and we thank the reviewer for identifying it. The abstract has been modified accordingly.

Finally, just out of curiosity, antagonism wasn’t studied. Why not? I would love to see how your framework responds to antagonistic interactions. I would expect stratification (either concentric or columned) to perhaps break completely under many conditions. I understand this is clearly a case of “beyond the scope of this paper”, but perhaps explain at least why it is missing?

**Answer**

We have focused on the effect of resource availability over ecological interactions and therefore community assembly. *Antagonism/amensalism* has an inverse trend function of substrate concentration (inhibitory effect) than to the selected ecological interactions (*neutralism, competition* and *commensalism*). In this work the objective is to analyse the combination of two ecological interactions in which one has a positive impact and the other negative. We have chosen commensalism as the positive ecological interaction, and competition as the negative. The influence of substrate inhibition will present a similar trend than competition. As we are not quantifying this ecological interaction but analysing their trends and impact on community assembly, we have considered that the inclusion of *antagonism/amensalism* (a second negative ecological interaction) won’t change tendencies and it will be difficult to differentiate between the two negative interactions. . For these reasons, we decided to not include *antagonism/amensalism* in this study. A justification of why we decided to study these three ecological interactions has been included in S1 Appendix (*Decision of the eco-interactions*).

2. The results / discussions are dominated by results, and very thin on discussion. I would love to put these results in light of more data (interactions ranges by Alma Dal Co, mutational dynamics by Diane Fusco), and to also elaborate a bit more on how these colony dynamics (mostly derived from lab results) could translate to microbes leaving on a grain of soil, where there is much more lysis through phage predation (whereas your model, doesn’t take into account lysis at all).

**Answer**

We have complemented the discussion with a more detailed comparison with the available literature on *in vitro* and *in vivo* works. However, the interaction ranges studied by Alma al Co were considered out of the scope of this paper. Mutualism is not considered in this paper and adding mutational dynamics, dispersion of microbes or lysis through phage predation will just add complexity to the model that we cannot experimentally validate.

Plus, there’s a variety of other stressors in nature that a lab cannot possibly capture (nor should they try to, this is what models like yours are for!). This is not to say I would encourage to change the model, but just to make it very clearer what you did *not* do. This will inspire ideas for future work, which I think is important in discussion sections.

**Answer**

A new section has been included in the manuscript to refer to other stressors that control the spatial distribution of microbial populations, with a special emphasis on shear stress (detachment).

3. I find the link with Sara Mitri’s work interesting, but it requires much more unpacking. Neither 100mM nor 10mM give a “well-mixed” result, but I suppose your main point is to say they are not stratified. Acknowledging this may actually make the result more, not less interesting. Mitri really shows “well-mixed” outcomes, while your simulations still show something I would call far from “randomly distributed”. In Mitri’s work, resources are assumed to be truly mixed (if I remember correctly). Have you tried doing to same to confirm that particular result? If so, it would be a great addition to the supplement.

**Answer**

The main comparison of our results with Sara Mitri’s work (Mitri *et al.* (2016), *Resource limitation drives spatial organization in microbial groups*) is to point that regardless the ecological interaction established in the system, a stratified structure is generated (obtaining the same spatial organization than neutralism situation). With that, we are able to identify *neutral environment* as transient, because limitation of substrates will eventually emerge if diffusion resistance through the aggregate is occurring. Initializing the simulations with a higher inoculum and without diffusion resistance of substrates through the aggregate, we obtained a higher intermixing of microbial populations. A Supplementary Figure (S1 Fig) has been added to confirm the results of Mitri *et al*. (2016).

I do appreciate the key point here, in that there is no difference between the 3 interaction modes, but I would really suggest to not call this “randomly distributed”, as they still clearly form spatially separated groups.

**Answer**

Instead of calling randomly distributed populations, we rephrased to “[…] *no particular spatial arrangement of microbial populations was observed in any of the considered eco-interactions*” (Lines 151 – 152) .

4. I really enjoy the final results (Fig 6 and 7), but I am wondering to what extend you could explain this without taking O2 into consideration.

**Answer**

Without taking O_2_ into consideration Figs. 6 and 7 will change. In that situation, only commensal environment would be present, and the results will be analogous to Fig. 5.

Can we see layered vs. columned stratification when *not* changing between two limiting resources (O2 and resource A), but simply modifying other parameters? Looking at equation 1 (for the eco-interaction modulus), I feel like the same differentiation of layered vs. columned stratification can be found by playing with the diffusion constant (D) and the concentration (C), giving either a $\phi_{EI}$ greater than 1, or smaller than 1. Perhaps the rates you’d have to assume for this are unreasonable biologically, but in that case that is also very interesting to mention at in the text, as that would imply that these differential patterns cannot be explained by a single limiting nutrient.

**Answer**

Indeed, we could see this transition from layered to columned stratification and vice versa modifying other parameters, for example the diffusion constant of substrates. A new section has been added to mention the influence of the diffusivity of substrates on spatial distribution of microbial communities with an example to illustrate it (S9 Fig). We simulated again one of the cases of concurrence commensalism (substrate A) and competition (O_2_) but now reducing the diffusion coefficient of substrates A, B, C and D (from 3.60x10-6 m2/h to 0.5x10-6 m2/h). As example, the environment with 1.0 mM of A and 10.0 mg/L of O2 (competitive environment, $\phi_{EI}$ = 0.65) was applied starting with the same inoculum. With the new diffusion coefficients, substrate A (instead of O2) was the most limiting, obtaining a layered stratification of microbial populations (commensal environment, $\phi_{EI}$ = 1.51).

5. The concept of the dynamical fitness landscape is introduced in the introduction, but there is no further reference to it. Although I can see that your model fits this concept, it is not actually adding anything to this theory explicitly. Similar to point 2, perhaps discuss it some more later, or, not mention it in the introduction at all.

**Answer**

*Dynamical fitness landscape* concept (with emphasis on the reciprocal organism–environment influence on microbial community assembly) is mentioned in the Introduction to present the *ecological environment* concept, which encompasses not only reciprocal organism–environment influence, but also the organism–organism influence. Proper modifications in this part of manuscript have been made to emphasizes the reformulation of *dynamical fitness landscape* concept adding the organism–organism influence, defining the *ecological environment* concept.

6. The GitHub link in the methods was broken, so I couldn’t actually confirm the published code is presented clearly. As someone who spends a lot of time trying to install tools, I would appreciate it if I can have a look to confirm everything is in order.

**Answer**

We tested the link in the *submission file* and the hyperlink addressed to the code and now they work correctly. Additionally, a proper README file has been written in order to ensure that any user (familiarized or not with MATLAB) is able to get the code up and running.

**Minor points/Typos:**

1. This is subjective, but I find “eco-interactions” to be an unnecessary abbreviation of ecological interactions.

**Answer**

In this case, the abbreviation of ecological interaction to “*eco-interactions*” was implemented from the beginning of the manuscript to later be used in the theoretical modulus presented in this study – the Eco-interaction modulus ($\phi_{EI}$), instead of “*Ecological interaction modulus*”. For this reason, we decided to keep this abbreviation on the manuscript.

2. L37. Typo (reveals)
3. L69. Typo (ammonia-oxidizers)

**Answer** (points 2 and 3)

All these typos have been corrected, and the manuscript has been carefully reviewed again.

4. L69. I think I have some more issues with that sentence. I know what is said, but I find it hard to parse. I’m not even sure it is grammatically correct (?).

**Answer**

This sentence has been rewritten.

5. L89. Our results suggests that ecological environment would control spatial organization -> no surprise. What is interesting about your model is not *that* is shapes spatial patterns, but *how* it shapes is.

**Answer**

This sentence has been modified.

6. L124. Protection only considers two species?

**Answer**

*Protection* case has been removed from the study. Instead, *in vitro* and *in vivo* examples have been referenced to highlight the ecological impact of *layered stratification*.

7. L296. Not sure if the word “even” in this sentence is necessary or clarifying at all.

**Answer**

Accordingly, the word “*even*” has been removed.

8. L235. I had to dive into the stoichiometry to figure out why B2 and B3 have different growth dynamics. I think you may want to introduce here that B2 grows on B1’s products, and B3 on B2’s products, because as you phrase it now there is too much stress on “how the kinetics are identical”, which makes this result confusing for the reader. (this was one of my major points, but this sentence here is an example where the confusion arises)

**Answer**

This minor point has been solved and answered in Major Point 1.

9. L337. The word concentric is mentioned here, but nowhere else in the text. For me it actually would have helped if you coined this earlier, because I had to keep looking back at the figures to understand layered vs. columned stratification, while the word “concentric” would clear that right up.

**Answer**

Totally agree. We have introduced the term “concentric” to the description of spatial distributions of substrate-related commensalism case.

10. L340. This sentence makes it sound like nobody believes the environment controls these patterns at all. I would rather read “[…] the environment has a significant impact on spatial distribution patterns as observed in nature.”, or something along those lines.

**Answer**

The intention of this sentence is to highlight our capacity to control the community assembly through the environment (or conditions) of the system. Some clarification to this has been added and the sentence has been rephrased.

11. Figure 3B. It’s unclear what the three statistical groupings are. The caption explains this, but I would suggest adding a line as is common practice.
12. Figure 4. Same layout as three, so same remarks.

**Answer**

We have changed the statistical significance level is presented in Fig. 3B, 5B and S6. The mentioned description in these captions has been removed.

13. L370. I wasn’t immediately sure what R(x,y,t) did, only when I got to line 379. The introducing sentence may need further unpacking (“A term of reaction is added function…”), as the grammar is also awkward.
14. L379. Alright, so I understood now. You may want to add to this section that you are assuming steady-state for the production/degradation, etc. ◊ You do mention this later in L421, but I find it may help to mention this also here.

**Answer** (points 13 and 14)

The description of reaction term R(x,y,t) has been restructured and revised in order to improve its comprehension, clarifying how it participates in the model.

15. L390. Please make explicit that you assume no stochastic death (only through shrinking) and assume no lysis of cellular “products”.

**Answer**

The description of death/inactivation of cells is described in Methods (Description of the mathematical model; Lines 482 – 485; and 507 – 509), making explicit that no stochastic death was assumed. The no consideration of cell lysis was included in Method section.

16. L446. Is the word “the relationship” missing in the last part of this sentence? Otherwise, not sure what this sentence means.

**Answer**

In order to clarify this section of Methods, *“cell metabolism”* have been replaced by “*their metabolic stoichiometries*” (Line 540).

17. L449. For clarity, is the initial distribution the only source of stochasticity in the model, or is there noise on other processes?

**Answer**

No, it is not. More stochasticity in the model was assumed: (*i*) the initial mass of a new cell is a random percentage of the total mass of the parent cell (Fig 9C; Methods: Lines 507 - 509); (*ii*) when a cell divides, a random position for the new individual in the neighbourhood of its parent is assigned (Methods: Lines 510 – 514).

18. L476. Please explain the variables in the order they appear in equation 11-13, this is very confusing.

**Answer**

A reorganization of the description of the variables in Equations 11 – 13 has been carried out.

19. SupS1. Check grammar on page 12, “Due to bacteria will not move very far from original position, it is assumed that chosen neighbouring bacteria stay constant”

**Answer**

This sentence has been checked and corrected. S1 Appendix has been also carefully reviewed. Note that modifications in Supplementary Material (S1 Appendix) have not been highlighted because this must be submitted in the final format (no post-edition is performed).

We would like to thank **Dr. Bram van Dijk** for the provided suggestions which have helped us greatly to clear up and improve significantly the quality of our manuscript.

**Reviewer #2:**

**Major remarks:**

1. Radial and concentric organisation of microbial populations depending on ecological interactions, and the concept that ecological relationships and the environment interact to shape the ecosystem, are not novel findings. These are already modelled in detail in, for example, reference [20] (Momeni et al.). The paper needs more comparison with existing models, such as [19] (Xavier et al) and [20] (Momeni et al). In particular, the authors should investigate what distinguishes their model from these previous models, and what new insights they have gained.

**Answer**

A literature review of existing models and studies focused on the spatial organization of microbial populations has been added with the corresponding comparison to our results. We have rewritten the last section of Introduction to clarify the main aim of our study (Lines 85 – 103. Additionally, the concurrence of multiple eco-interactions (Lines 292 – 383) and shear stress impact were also evaluated (Lines 384 – 416).

2. There should also be additional comparison in the discussion of the new insights with the available literature on *in vitro* and *in vivo* biological aggregate communities. Which of the observed patterns have previously been observed *in vivo* or *in vitro*? Is there an unexplained pattern that this model can explain?

**Answer**

The discussion has been complemented with a more detailed comparison with the available literature on *in vitro* and *in vivo* works (Lines 166 – 170; 220 – 222; 283 – 291; 303 – 305; 336 – 344; 362; 492 – 494; 415 – 416).

3. It is unclear how the visualisations or statistics are performed with respect to the replicates in, for example, Fig. 3B and fig 4D. Are all fitness measurements from each replicate combined for each species? This seems to me to violate the assumption of independent observations. Multiple fitness values derived from the same replicate cannot be considered independent observations. This should either be corrected, or the reasoning should be described.

**Answer**

In order to not violate the assumption of independent observations, fitness figures (Figs. 3B, 4B, 5B and S6) has been corrected appropriately. The median of microbial fitness has been calculated for each replicate before the visualization and statistical analysis (see Figure captions).

For the relative colony size analysis (Fig. 4D), each microbial colony was treated as independent observation because in the competition case the concentration of substrate is fixed, and the microbial fitness of all species is the same (Fig. 4B). All figure captions have been accordingly modified and the treatment of each microbial colony as independent observation has been argued in Method section (*Statistical analyses*).

4. A possibly related question: How do you explain the very strongly significant differences between some species in the neutral condition of Fig. 3B? It seems to me that any differences between species in the neutralism condition would be the result of stochastic variation and should not be so strongly significant. This may be the result of treating related observations as independent. In addition, if the variation between replicates is high, more than three replicates should be performed per condition.

**Answer**

This question is already addressed in the manuscript (Lines 188 – 199). Certainly, stochasticity is present in the model (distribution of species in inoculum, position of the new cell around parent cell and mass of the new cell and parent). Differences between microbial populations (Figs 3A and 3B) and the observed correlation between relative abundance and microbial fitness (Fig S3A), highlight the importance of the position of individuals in the aggregate. Competition for space is inherently present in aggregates as long as there is not substrate saturation. This part of the manuscript has been accordingly extended to highlight the presence of competition for space in aggregates (Lines 197 – 199).

5. There are references to an implementation of the “protection” eco-interaction in the text, but no results outside of the single run of figure S8. This condition should either be expanded upon or be removed from the text if it is not analysed in any depth.

**Answer**

*Protection* has been removed from the study. Instead, *in vitro* and *in vivo* examples have been referenced to highlight the ecological benefits of *layered stratification* (Line 348). With that, we are partially addressing the **Major point 2** as well.

**Minor remarks:**

1. It is unclear at what point in the simulation measurements were taken in Fig. 3C. At line 108 you state that aggregates grow until steady state is reached, but you do not state when this is reached in Fig. 3, or if this is reached in Fig. 2. If both are in steady state, why is the shape of the colony for the 1mM condition of Fig. 3C very different from that in Fig. 2C for the same condition?

**Answer**

The missing information about when the aggregate images were captured (*steady state time*) have been included in the caption of the figures (Figs. 3C, 4C, 5C, 6 and 7). All simulations presented in Fig. 2C were not in steady state. Due to this, the shape of the colony for 1.0 mM condition of Figs. 2C and 3C are very different. This have been clarified in the caption of Figs. 2 and 3C. The figures are not in steady state, to show the transition from saturation of substrate/s inside the granule (100 mM) to limitation (1.0 mM), and also to state the association between the radial expansion of populations and the presence of gradients of substrate concentrations (S1 Video and S2 Video).

2. The description of the model at line 98 is insufficient to understand the subsequent results without also reading the methods - particularly the ‘colony size’ referred to frequently should be defined around line 98.

**Answer**

To improve the comprehension of the results, we have clarified the definition of ‘colony size’ in this part of the manuscript (Lines 231 – 232 and caption of Fig 4). The aim of the brief description of the model in Result/Discussion was to give a rough idea the scope and the boundaries of the model with an expansion in supplementary. This has been slightly modified to increase the reader’s understanding.

3. What is colony size relative to in Fig. 4D?

**Answer**

Colony size is relative to the total perimeter of the aggregate. To address this comment, we have improved the caption of Fig. 4D adding the missing information about Relative colony size.

4. A link to the code is included, but the readme is so brief that it remains unclear to me how I should run the code, as someone not very familiar with MATLAB.

**Answer**

A README file has been expanded and revised in order to ensure that any user (familiarized or not with MATLAB) is able to get the code up and running.

5. Line 260 - You introduce competition for O2 here as if that was already a factor, but you have not mentioned it previously.

**Answer**

To address this comment, the mentioned line and *Set of simulations* section on Methods have been modified (Lines 296; and 538 – 548). Instead of considering the presence or absence of O_2_ as a factor, we have just included the concurrence of multiple eco-interactions as a case (commensalism + competition) together with neutralism, competition and commensalism. Figure 10 has been modified accordingly.

6. How exactly do you determine when/if steady state has been reached? Would the results be greatly altered by placing the elsewhere? What is the variability between runs and conditions in reaching this steady state?

**Answer**

In the section Results/Discussion we have addressed the definition of steady state (Lines 122 – 124). In practice, all simulations were running longer times than the placed *steady-state time* to be on the safe side.

**Typos:**

7. The paper is generally well-structured but needs additional editing for grammar and spelling throughout the paper. Some examples:

Line 37: revel → reveal

Line 38: dictates → dictate

Line 59: are → is

Line 69: oxidizes → oxidizers

Line 70: eco-interaction → eco-interactions

Line 89-91: I do not understand the use of “would” here, the model indicates this process already occurs.

Line 118: - You mean produced here, not metabolised

Fig. 1: Ammensalism → amensalism

**Answer**

All these typo mistakes have been corrected, and the manuscript has been carefully reviewed again.

We would like to thank **Reviewer #2** for the suggestions provided. They have helped us greatly to clear up and to significantly improve the quality of our manuscript.

**Reviewer #3:**

**Minor comments:**

1. Figure 1: Only three out of the six possible ecological interactions are studied in this manuscript (even without counting the many more combinations arising from concurrent interactions). I think it is fine to focus only on the three cases discussed in this manuscript; however, it would be helpful to include either a justification of why these three are chosen (perhaps in the introduction), or a brief description/speculation of what happens in the other cases (perhaps in the discussions).

**Answer**

Considering *neutralism* [0,0], *competition* [–,–], *commensalism* [0,+], *mutualism* [+,+], *parasitism* [–,+] and *amensalism* [–,0] as the 6 main ecological interactions (Fig. 1), this study has focused only on the three first cases (*neutralism*, *competition* and *commensalism*) and concurrence of *competition* and *commensalism*. Here, we stated the reasons of this decision:

- *Mutualism* [+,+]. Distinguishing cross-feeding (or *syntrophy*) and co-protection (or *symprostasy*) as two specific ecological interactions belonging to mutualism:
  - Cross-feeding (*substrate-related mutualism*). This ecological interaction has already been studied by Mitri, S. *et al.*, 2011 (doi: 10.1073/pnas.1100292108) and Momeni *et al*, 2013 (doi: 10.7554/eLife.00960), evaluating the influence of substrate(s) concentration and the presence of competition between microbial communities. Additionally, *mutualism* is one of the less frequent ecological interactions among culturable bacteria (only 5% of all assessed interactions among 20 soil bacteria across 40 carbon environments corresponded to a mutualistic interaction; study from Kehe *et al.*, 2021, *Positive interactions are common among culturable bacteria,* doi: 10.1126/sciadv.abi7159). As stated in Palmer and Foster, 2022 (*Bacterial species rarely work together*, doi: 10.1126/science.abn5093) – “*Negative interaction prevails, and cooperation, where two species both benefits, is typically rare*”.
  - Co‑protection (*inhibitor-related mutualism*). From an ecological perspective, the resource availability has a direct impact on interactions among species (e.g., a limited resource environment intensifies the competition for this resource). For this reason, the influence of substrate concentration has been evaluated in this study. Note that when inhibition of some community member is present in the system (either for presence of inhibitor in the environment, (*co)protection*; or produced by other community member, *amensalism*), the inhibitory influence is strengthened as higher is the concentration of inhibitor. Therefore, co‑protection has an inverse substance-related trend to the selected ecological interactions (*neutralism, competition* and *commensalism*). For this reason, we decided to not include co‑protection in this study.
- *Parasitism* [–,+]. One of the aims of this study is to study the combination of two ecological interactions in which one have a positive impact (as *commensalism*) and the other a negative impact (as *competition*) (Line 100). Due to *parasitism* has already a negative impact in one of the species, we decided to not include it in this study.
- *Amensalism* [–,0]. As (co)protection, *amensalism* has an inverse substance-related trend to the selected ecological interactions (*neutralism, competition* and *commensalism*). Moreover, *amensalism* has already a negative impact in one of the species. For these reasons, we decided to not include *amensalism* in this study.

Highlighting (in bold) of the considered ecological interactions in Figure 1 have been removed. Instead, the justification of why these three ecological interactions has been included in S1 Appendix (*Decision of the eco-interactions*). Additionally, new sections describing what happens in other cases have been included in the manuscript to complement the discussion.

2. Line 169: Please define “mass transfer resistance” in this context.

**Answer**

A short clarification of “mass transfer resistance” has been included in manuscript (Lines 190 – 191).

3. Figure 2: I think it would be helpful to show examples of the initial distribution of cells (i.e., the inoculum) in the figure as well.

**Answer**

An example of the initial distribution of cells (inoculum) has been included in Fig 8A (*Methods – Description of the mathematical model*).

4. There are existing examples of three-species spatial patterning (simulations and experiments) in (Breugelmans et al., FEMS Microbiol Ecol, 2008, DOI:10.1111/j.1574-6941.2008.00470.x), and (Momeni et al., eLife, 2013, DOI:10.7554/eLife.00960), which I think are in line with authors’ findings and can be mentioned as supporting evidence. I think, an expansion of the contributions in prior work (many of them cited in the introduction) would have been nice (optional).

**Answer**

Appropriate expansion of prior contributions (including Breugelmans *et al*. (2008) and Momeni *et al.* (2013) has been included to support our results and complement the discussion.

5. Related to Figure 2, the “layered stratification” as described in the text is not immediately obvious when visually examining the patterns in Fig 2. This is perhaps also related to the previous comment in which existing examples more clearly exhibit “columned stratification” and “layered stratification”, when the cross-sections of communities are examined. While I understand that the authors in the context of microbial aggregates have focused on the (x,y) patterns in this manuscript, perhaps the terminology can be reconciled and justified by mentioning the relation with the (x,z) coordinates.

**Answer**

We assumed that this comment is related to Fig 5 instead of Fig 2, because “*layered stratification*” term was not introduced until the description of spatial distribution of communities in commensalism case (Fig 5C). In this case, we improved the description of spatial distribution of microbial communities in commensalism by adding the term “*concentric disposition*”.

6. Line 247: I would suggest not using the term “evolution” to describe changes in community properties during the emergence of spatial organization to avoid confusion.

**Answer**

Term “*evolution*” has been replaced avoiding confusions and highlighting that we are talking about the change of growth rate in time.

7. Lines 396, 632, and 634: Again, the term “evolution” is used in a context that might cause confusion

**Answer**

Term “*evolution*” has been replaced to avoid any confusion.

8. I don’t think the growth rate comparisons at different resource levels in Fig 5D are fair, since the amount of growth (say, quantified as the number of generations of total population growth) is very different at different [S]_T_ levels

**Answer**

It is true that the substrate concentration has an impact on population growth. For this reason, in Fig 5D growth rate ratios (${\tilde{\mu_{B2}}}/{\tilde{\mu_{B1}}}$ and ${\tilde{\mu_{B3}}}/{\tilde{\mu_{B1}}}$) instead of the actual growth rate of population (*µ_B1_*, *µ_B2_* and *µ_B3_*) was employed to elude any influence and, therefore, any possible noise in our analysis. A clarification about the use of growth rate ratio instead of actual growth rate of population was added in Fig 5D caption. Additionally, an introductory description of growth rate ratio was added in the description of Fig 5D, which was missing in the previous version.

9. The link to the codes should be: https://github.com/Computational-Platform-IbM/IbM (it's written correctly, but the hyperlink points to the wrong address).

**Answer**

We tested the link in the *submission file* and the hyperlink addressed to the code and now they work correctly.

We would like to thank **Reviewer #3** for the suggestions provided. They have helped us greatly to clear up and to significantly improve the quality of our manuscript.
