# Supplementary material for: Environmental and ecological controls of the spatial distribution of microbial populations in aggregates
Source: PLoS Comput Biol. 2022 Dec 19;18(12):e1010807. doi: 10.1371/journal.pcbi.1010807 (PMC9810174; doi:10.1371/journal.pcbi.1010807)
Supplement: Attachment — Submitted filename: Letter-referees_EMartinez-Rabertetal2022.docx [file pcbi.1010807.s021.docx]

Glasgow, 8^th^ December 2022

Dear Editors,

Please find enclose the detailed answers to the comments that referees have made, and the revised version of the manuscript “*Environmental and ecological controls of the spatial distribution of microbial populations in aggregates*” by Eloi Martinez-Rabert, Chiel van Amstel, Cindy Smith, William T. Sloan and Rebeca Gonzalez-Cabaleiro for publication in *PLOS Computational Biology*. As indicated, two versions of the revised manuscript have been submitted: one with all revised portions of the manuscript highlighted in red font; and other without highlighting the modifications (clean version).

We believe we have been able to address all the reviewer comments positively and that we have modified the manuscript substantially addressing all the concerns reviewers had. We like to thank all the reviewers for their valuable inputs that have helped us to improve our manuscript substantially.

We hope the manuscript can now be accepted for publication in *PLOS Computational Biology*.

Yours sincerely,

MSc Eloi Martinez-Rabert

Advanced Research Centre (ARC),

Chapel Lane, Glasgow, G11 6EW

*2424069M@student.gla.ac.uk*

**Answers to the Reviewer of the Manuscript:**

**“Environmental and ecological controls of the spatial distribution of microbial populations in aggregates”**

We appreciate the comments of the reviewers and believe their suggestion have allowed us to improve the quality of our manuscript. Please find our replies to the reviewer’s comments in the following section.

**Reviewer #1:**

*You went above and beyond with this revision. I have recommended this manuscript for publication.*

**Reviewer #2:**

**Major remarks:**

1. I do not understand your explanation for treating each microbial colony as an independent observation in fig 4D. These differences in colony size must be a result of the (indirect) interactions with other colonies. Treating them as independent overestimates your number of truly independent observations. I would expect each run to produce a single number (mean colony size), and for these to be compared, or for some other statistical method to be used that takes into account that observations are not independent.

**Answer**

In order to not violate the assumption of independent observation, the figure of relative colony size (Fig. 4D) has been corrected appropriately. The average of relative colony size has been calculated for each replicate before the visualization and statistical analysis (see Figure caption). The data has been represented through individual data points following the Minor remark #5. The section of Methods *Statistical analysis* has been modified appropriately (Lines 590 – 595).

2. Similarly, I am missing an argument for the independence of observations in figures such as 3B. I would instead argue that the population of one species cannot be considered independent from that of another species in the same simulation, due to competition for space, and so cannot be compared using this test. A paired t-test would be more appropriate for this type of observations.

**Answer**

In order to not violate the assumption of independent observation, the paired *t*-test has been used to calculate the statistical significance between microbial fitness of B1, B2 and B3. Fitness figures (Figs. 3B, 4B, 5B and S7) and the section of Methods *Statistical analysis* (Lines 590 – 595) have been modified appropriately.

If results remain significant for the neutralism condition, some explanation would be good on why there are three significant pairwise interactions out of 9. It may be be purely stochastic variation, of course, but I would urge you to make sure the initial seeds are different, and there is no bug in your code leading to species 2 and 3 being favoured. (I would not expect the different statistical methods to change the significance of any other results - they seem quite clearly different to me from the plots).

**Answer**

After performing the paired *t*-test for neutralism conditions, microbial fitness on 1.0 mM case remained significant. The reason of this is already discussed in the manuscript (Lines 189 – 200). We tested the creation of initial seed, and they were completely random.

3. The statistics performed in figure 5 are unclear to me. What is the meaning of the width of the lines under the stars? And what, exactly, is being compared there?

**Answer**

In Figure 5D is comparing the difference of growth rate ratio of active B2 and B3 (${\tilde{\mu_{B2}}}/{\tilde{\mu_{B1}}}$ and ${\tilde{\mu_{B3}}}/{\tilde{\mu_{B1}}}$, respectively) between the different concentrations of substrate (1.0 mM, 0.5 mM, 0.2 mM and 0.1 mM) over the simulation time (dimensionless time was applied to compare the simulations with different maximum time of simulation, t_max_). The width of the lines indicates the time in which correspond the significance level (represented with the asterisks). The meaning of the width of the lines has been included in the caption of Figure 5 to avoid any confusion. A restructuring of caption has been done as well.

Intuitively, I would expect the two lines to be compared, but you say in the caption that you are comparing the 1mM condition with the other conditions, which are all combined into a single line. It would be much clearer to represent each condition (or maybe only the 1mM and 0.1mM, for clarity) as its own set of two lines, especially if there are significant differences between them.

**Answer**

We are comparing the 1.0 mM conditions with the other (i.e., 1.0 mM vs 0.5 mM; 1.0 mM vs 0.2 mM; 1.0 mM vs 0.1 mM). This has been clarified in the caption of Figure 5.

4. I do not understand your conclusions on shear. Looking at S3Video (the figure is compressed a bit), it seems to me that layered stratification is still present in the commensalism and the commensalism+competition condition. While not as clear as in the no detachment condition, the average location of B3 populations seems closer to the centre than that of B2, which is closer than that of B1. This is of course only based on a single picture - your hypothesis may very well still be correct.

**Answer**

Although it is true that the metabolic successors are in average closer than B1 to the centre (especially B3), in S3 Video you can see that more B2 and B3 are on the external part of the aggregate with B1 than the simulation experiments with no detachment. Moreover, it is only a matter of time before the last metabolic successor (B3) being just next to B1 and B2 on the external part of the aggregate (some B3 cells have already reach the external part of the aggregate).

It would be good to have a few runs and some statistical analysis of this behaviour - it may also depend on the initial configuration.

**Answer**

In addition to running triplicates when detachment was considered, we used the same inoculum for both situations (considering or not detachment) to avoid the putative influence of the initial configuration. This is now indicated in the captions of Fig 8 and S3 Video (see response to Minor remark #11).

5. The conclusion at lines 419-420 and 430-433 is stated too strongly - your results do not show that space plays a fundamental role in in vivo or in vitro microbial aggregates.

**Answer**

We replaced “*plays a fundamental role*” by “*influences*” (Line 424) and the last section of Conclusions (Lines 434 – 437)

6. I do not understand your conclusion that neutralism is a transient state (line 422). It implies that you do not consider your neutralism condition to be neutralism, as it does feature a competition for space. If this is the case, a different term should be used for ‘true’ neutralism, and neutralism with competition for space, to prevent confusion.

**Answer**

The term “*neutralism*” has been replaced by “*neutral environment*” to avoid any confusion (Line 426).

**Minor remarks:**

1. Your instructions to run the code are incorrect on a crucial point: 7. It says the user should run the code with “IbM(sim_xxxx)”, but the user should run “IbM(xxxx)”.

**Answer**

This mistake has been corrected.

2. S1 Fig is a good addition, but you should define the phrase “diffusion resistance”, as used in the caption, or use a different phrase.

**Answer**

Instead of “*diffusion resistance*”, we used “*substrate* *gradients*”, clarifying that in results S1B Fig we removed the substrate gradients.

Is the model simply well-mixed if this is disabled?

**Answer**

Although in the conditions of Figs. 2 and S1 (no substrate gradients inside the aggregate), there are still some properties of the aggregates (e.g., the shoving between cells). We could say that we are in the closest situation to a well-mixed system using the Individual-based Model.

In addition, it is unclear why the inoculum size is also changed between the two conditions, and whether these are at steady state or not.

**Answer**

We used a higher inoculum size to be as closer as possible to the simulations of Mitri *et al*. (2016). We stated in caption of S1 Fig that “*None of the the simulations are in steady state yet*”.

3. Line 94: You state that neutralism, competition, and commensalism are the most common bacterial interactions, but the citation (“Bacterial species rarely work together”) shows that, generally, it is competition, exploitation and amensalism that are the most common interactions.

**Answer**

Instead of saying that they “are the most common” bacterial interaction, we said that they “are more common than mutualism […]”

4. At line 122-124 you still say that all simulations are performed until a steady state is reached, but this is not the case for the first results you show (Fig. 2).

**Answer**

A clarification has been added to state that not all results we show are in steady state (Lines 124 – 125).

5. The visualisation with boxplots in, e.g., 3B is not ideal when n=3 or n=6 - plotting the individual data points would be more informative with such a low n.

**Answer**

Figs. 3B, 4B, 5B and S7 (visualization of microbial fitness with boxplots) have been modified according to this remark.

6. Line 176: you refer to Fig 4A, but I think you intend to refer to 3A.

**Answer**

This typo has been corrected.

7. The p-values in fig S3B do not match what you report at line 218.

**Answer**

The *p*-value reported at line 218 has been corrected. Additionally, the T-statistic instead of *p-*value was depicted in S3B Fig for [S]_T_ = 1.0 and 0.5 mM. This mistake has been corrected.

8. Line 429 and table S3: you still mention protective populations here, but nowhere else. In addition, the supplemental figures referred to in table S3 are misnumbered.

**Answer**

“Protective populations” are no longer mentioned in Conclusions nor Table S3. Numbering of supplementary figures of Table S3 has been corrected.

9. Line 508: what's the difference between a minimum mass and a negligible size?

**Answer**

In our model, the user can decide that if a cell reaches a size considered negligible (a smaller size than when this become inactive), the individual is removed from the system. This situation might occur if, for example, predation is considered. In this case, this setting is not active because predation was not contemplated. Then, the part “or size considered negligible” has been deleted from the text.

Also, what does it mean for a cell to be inactive? I assume it would not grow, but you mention in line 509 that it can become active when it increases in mass. How can this happen?

**Answer**

Indeed, when a cell becomes inactive (due to unfavourable conditions), this do not grow nor decay (mass of individual remains constant). Although the mass of individual does not change, the actual growth rate (µ) is still computed to detect if favourable conditions come back (i.e., µ > 0 again). In that case, the cell is reactivated. The transition from unfavourable to favourable conditions might occur by, for example, a perturbance on the system or a change in the community. “Inactive” term has been clarified (Line 512 – 513).

10. Scale bars are missing units in Fig. 8.

**Answer**

Units of scale bars has been included in Fig. 8.

11. How are random seeds determined? Line 542 implies that each simulation gets a different random seed, but in S3_Video the top three simulations have the same initial condition as the bottom three simulations.

**Answer**

In this case, the simulation experiments including detachment were started with the same inoculum as those without detachment to observe the genuine impact of the shear force to spatial distribution of microbial populations. This information has been included on the captions of Fig. 8 and S3 Video.

In addition, the section from line 538-543 claims all simulations are run in triplicate, but some are run six times.

**Answer**

This section of the manuscript has been modified to include those simulations that was run six times.

12. Editing for language is still needed - many lines have grammatical errors in them. Some examples:

70 "are collaborating" should be "collaborate"
101 "how ecological" missing "the"
102 "control" should be "controls"
153 There should be no comma here
166 "are" should be "is", or "structure" should be "structures"
284 “as” -> “such as”
302 “when concentration” - > “when the concentration”
398-399 “the same trend than Suarez et al al was observed”
422 “for” -> “of”
473-474 “one” (singular) and “are” do not match
478 "kinetics function" - should be "kinetics as a function", I think
504 "remained" should be "remaining"
550-551 This sentence is very hard to follow
576 "it was defined a"
739 no -> not

**Answer**

All these typo mistakes have been corrected, and the manuscript has been carefully reviewed again.

We would like to thank **Reviewer #2** for the suggestions provided. They have helped us greatly to clear up and to significantly improve the quality of our manuscript.

**Reviewer #3:**

*In my opinion, the authors have adequately addressed all reviewers' comments.*

1. The only remaining issue is that the link to the GitHub repository is still broken, but that might have happened during the pdf conversion. This can be fixed in the final production

**Answer**

We tested the link in the *submission file* and the hyperlink addressed to the code correctly.

We would like to thank **Reviewer #3** for the suggestion provided. It has helped us to ensure that the link to the GitHub repository works.
